# Supplementary material for: Post-Translational Modification Analysis of VDAC1 in ALS-SOD1 Model Cells Reveals Specific Asparagine and Glutamine Deamidation
Source: Antioxidants (Basel). 2020 Dec 2;9(12):1218. doi: 10.3390/antiox9121218 (PMC7761621; doi:10.3390/antiox9121218)
Supplement: Supplementary file 1 [file antioxidants-09-01218-s001.pdf]

Supplementary Materials

Post-Translational Modification Analysis of VDAC1 in ALS-SOD1 Model Cells Reveals Specific Asparagine and Glutamine Deamidation

SUPPLEMENTARY FIGURES

2	AcA^{2}VPPTYADLGKSARDVFTKGYGFGLIKLDLKTKSEN^{37}GLEFTSSGSA	47
48	NTETTKVNGSLETKYRWTEYGLTFTEKWNTDNTLGTEITVEDQLARGLKL	97
98	TFDSSFS^{104}PN^{106}TGKKNAKIKTGYKREHINLGC^{127}DVDFDIAGPSIRGA	141
142	LVLGYEGWLAGYQM^{155}NFETSKSRVTQ^{166}SNFAVGYKTDEFQLHTNVNDG	187
188	TEFGGSIYQKVNKKLETAVN^{207}LAWTAGN^{214}SNTRFGIAAKYQ^{226}VDPDA	231
232	C^{232}FSAKVNN^{239}SSLIGLGYTQTLKPGIKLTLSALLDGKNVNAGGHKLGL	277
278	GLEFQA	283

Figure S1. Map of the modified residues found in VDAC1 from analyzed NSC34 cell lines. The N-terminal acetylated alanine is shown in blue.

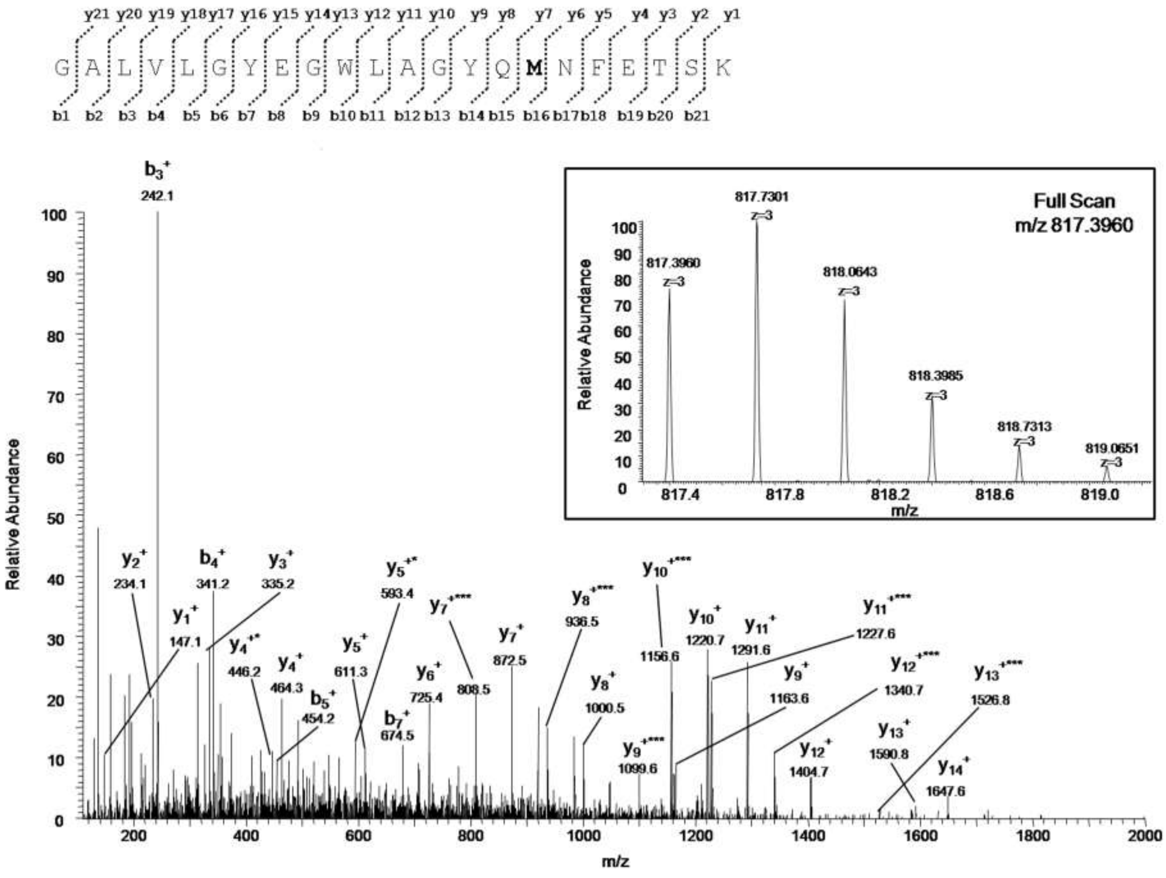

Figure S2. MS/MS spectrum of the triply charged molecular ion at m/z 817.3960 (calculated 817.3951) of the VDAC1 tryptic peptide from NSC34 cell line containing the methionine residue 155 in the oxidized form of methionine sulfoxide. The inset shows the full scan mass spectrum of molecular ion. Fragment ions originated from the neutral loss of H_2O are indicated by an asterisk. Fragment ions originated from the neutral loss of methanesulfenic acid (CH_3SOH, 64 Da) are indicated by three asterisks.

G[140] A L V L G Y E G W L A G Y Q **M** N F E T S K[161]

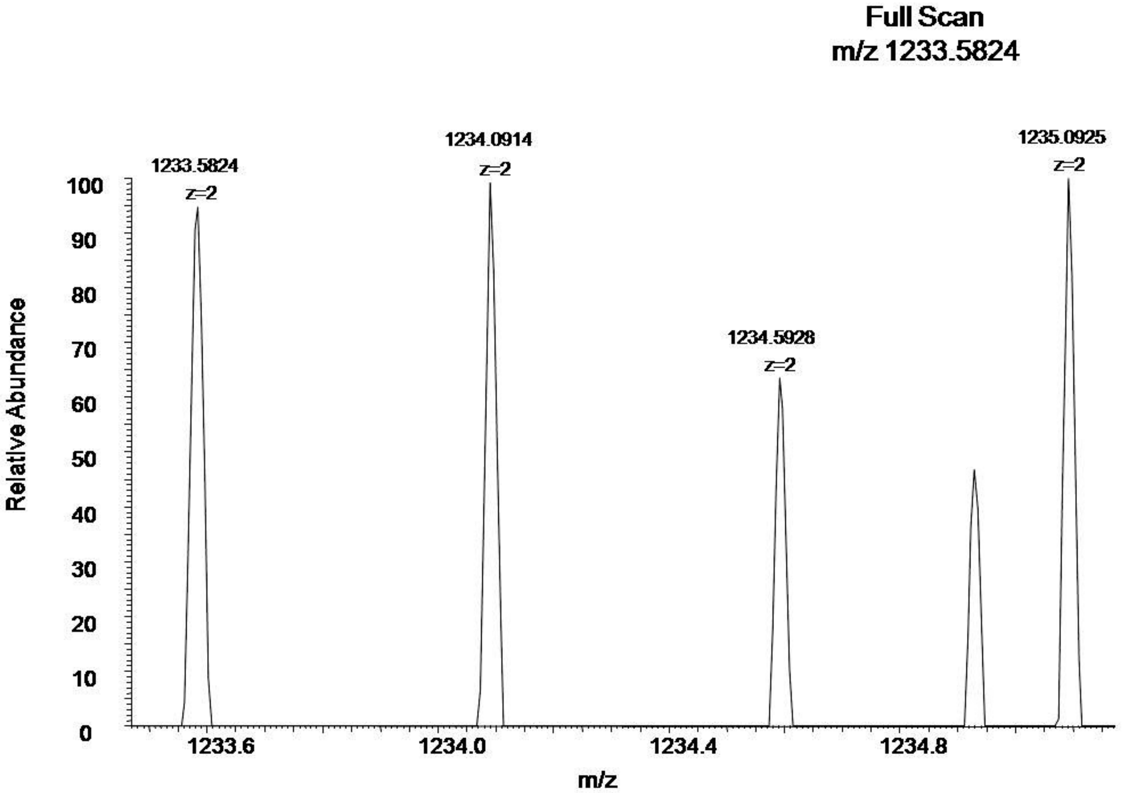

Figure S3. Full scan mass spectrum of the doubly charged molecular ion at m/z 1233.5824 (calculated 1233.5862) of the VDAC1 tryptic peptide from NSC34 cell line containing the methionine residue 155 in the oxidized form of methionine sulfone.

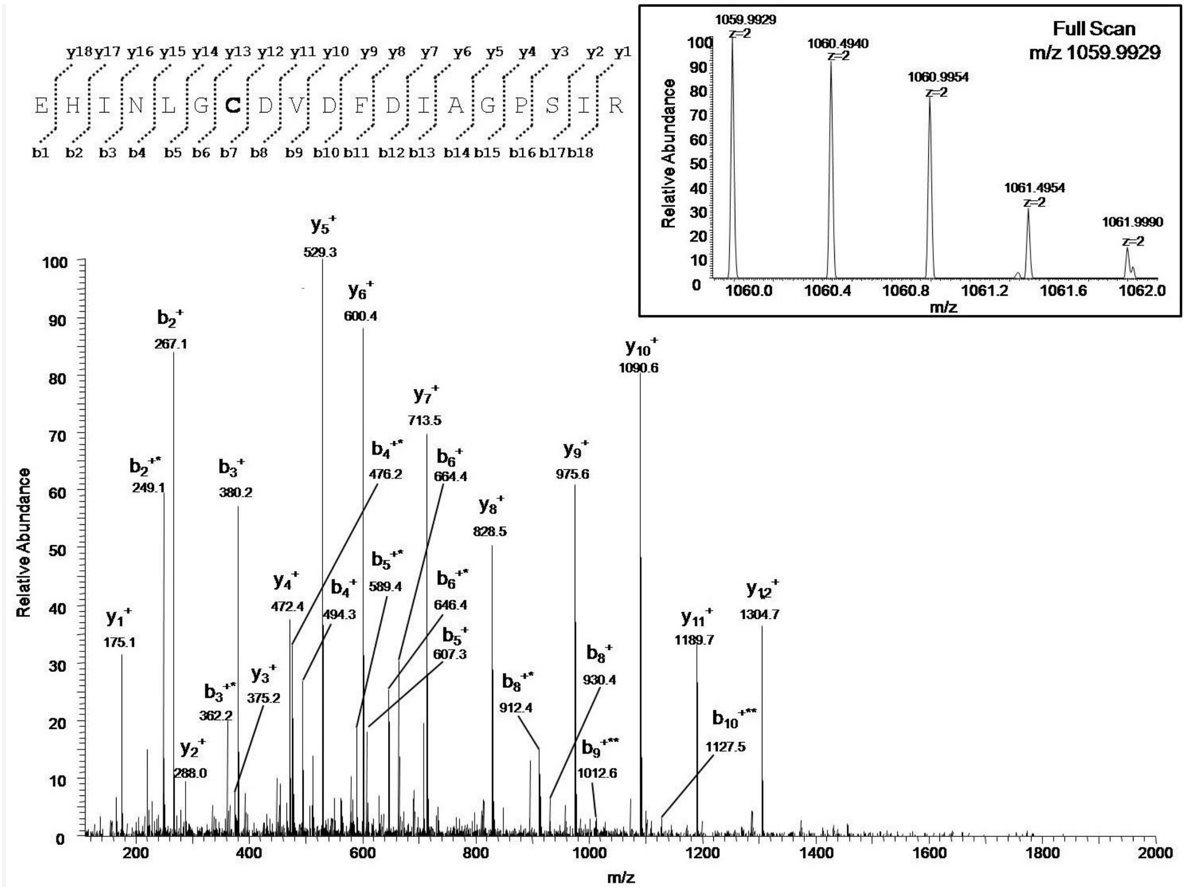

Figure S4. MS/MS spectrum of the doubly charged molecular ion at m/z 1059.9929 (calculated 1059.9919) of the VDAC1 tryptic peptide from NSC34 cell line containing cysteine residue 127 in the form of sulfonic acid. The inset shows the full scan mass spectrum of molecular ion. Fragment ions originated from the neutral loss of H_2O are indicated by an asterisk. Fragment ions originated from the neutral loss of NH_3 are indicated by two asterisks.

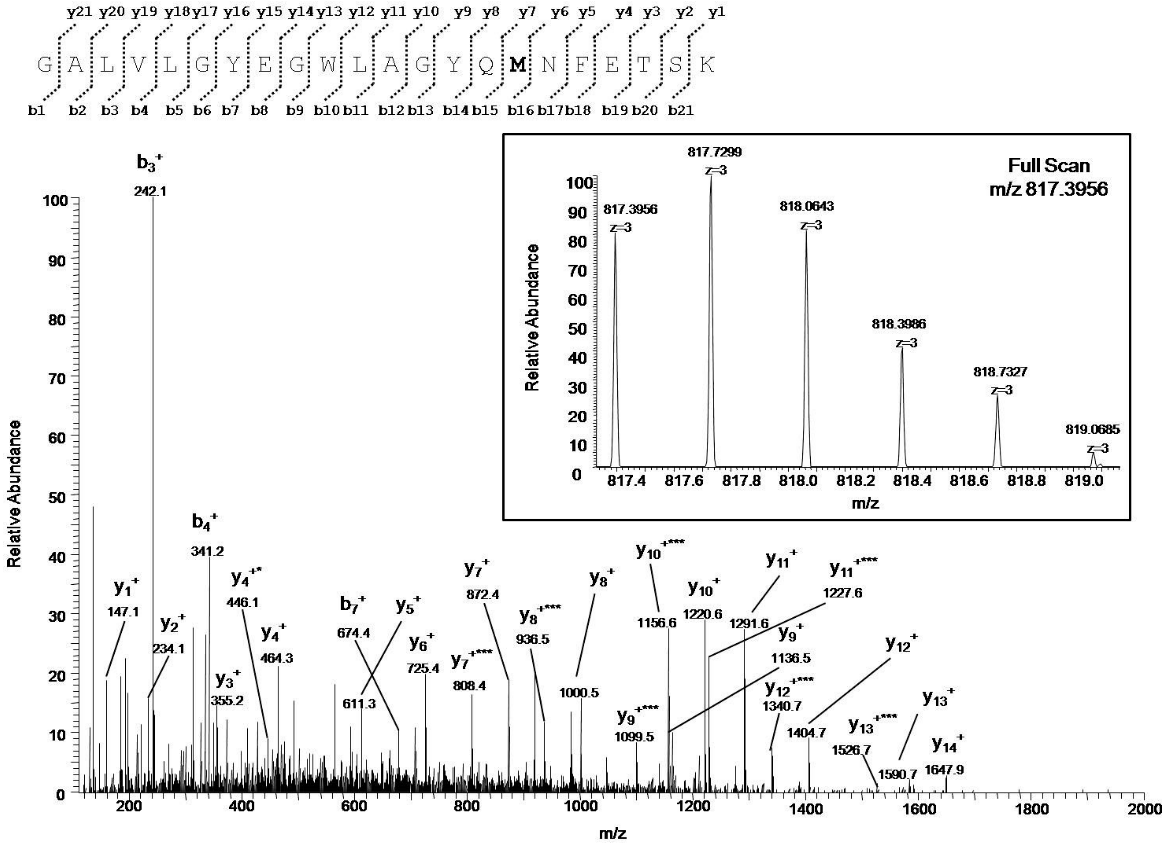

Figure S5. MS/MS spectrum of the triply charged molecular ion at m/z 817.3956 (calculated 817.3951) of the VDAC1 tryptic peptide from NSC34-SOD1WT cell line containing methionine residue 155 in the oxidized form of methionine sulfoxide. The inset shows the full scan mass spectrum of molecular ion. Fragment ion originated from the neutral loss of H_2O is indicated by an asterisk. Fragment ions originated from the neutral loss of methanesulfenic acid (CH_3SOH, 64 Da) are indicated by three asterisks.

G[140] A L V L G Y E G W L A G Y Q **M** N F E T S K[161]

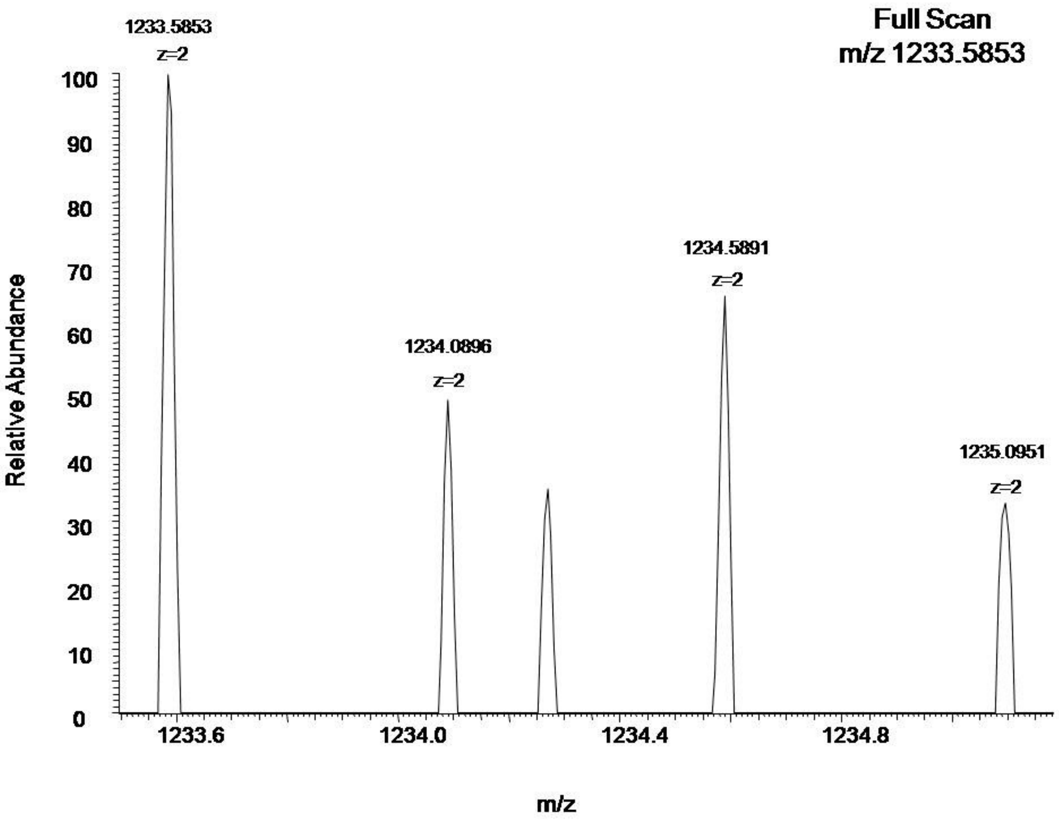

Figure S6. Full scan mass spectrum of the doubly charged molecular ion at m/z 1233.5853 (calculated 1233.5862) of the VDAC1 tryptic peptide from NSC34-SOD1WT cell line containing methionine residue 155 in the oxidized form of methionine sulfone.

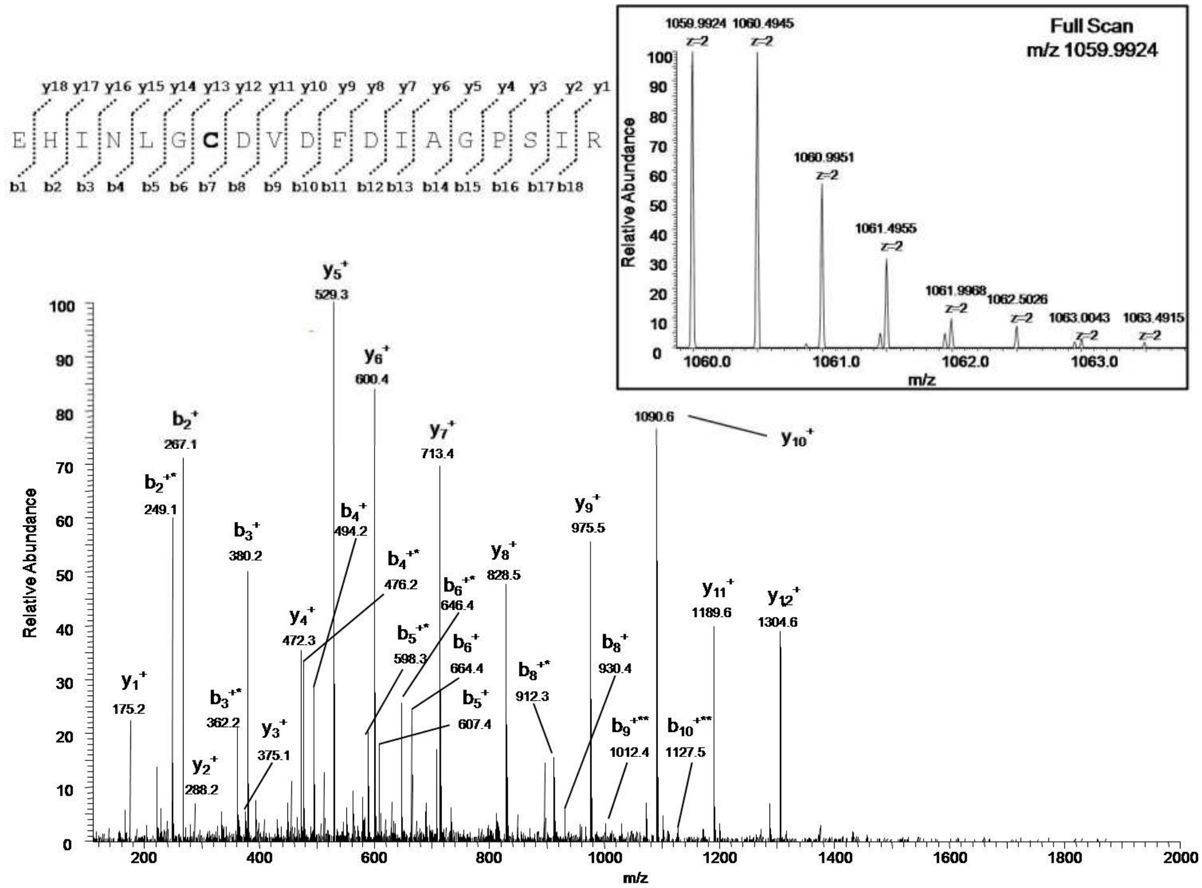

Figure S7. MS/MS spectrum of the doubly charged molecular ion at m/z 1059.9924 (calculated 1059.9919) of the VDAC1 tryptic peptide from NSC34-SOD1WT cell line containing cysteine residue 127 in the form of sulfonic acid. The inset shows the full scan mass spectrum of molecular ion. Fragment ions originated from the neutral loss of H_2O are indicated by an asterisk. Fragment ions originated from the neutral loss of NH_3 are indicated by two asterisks.

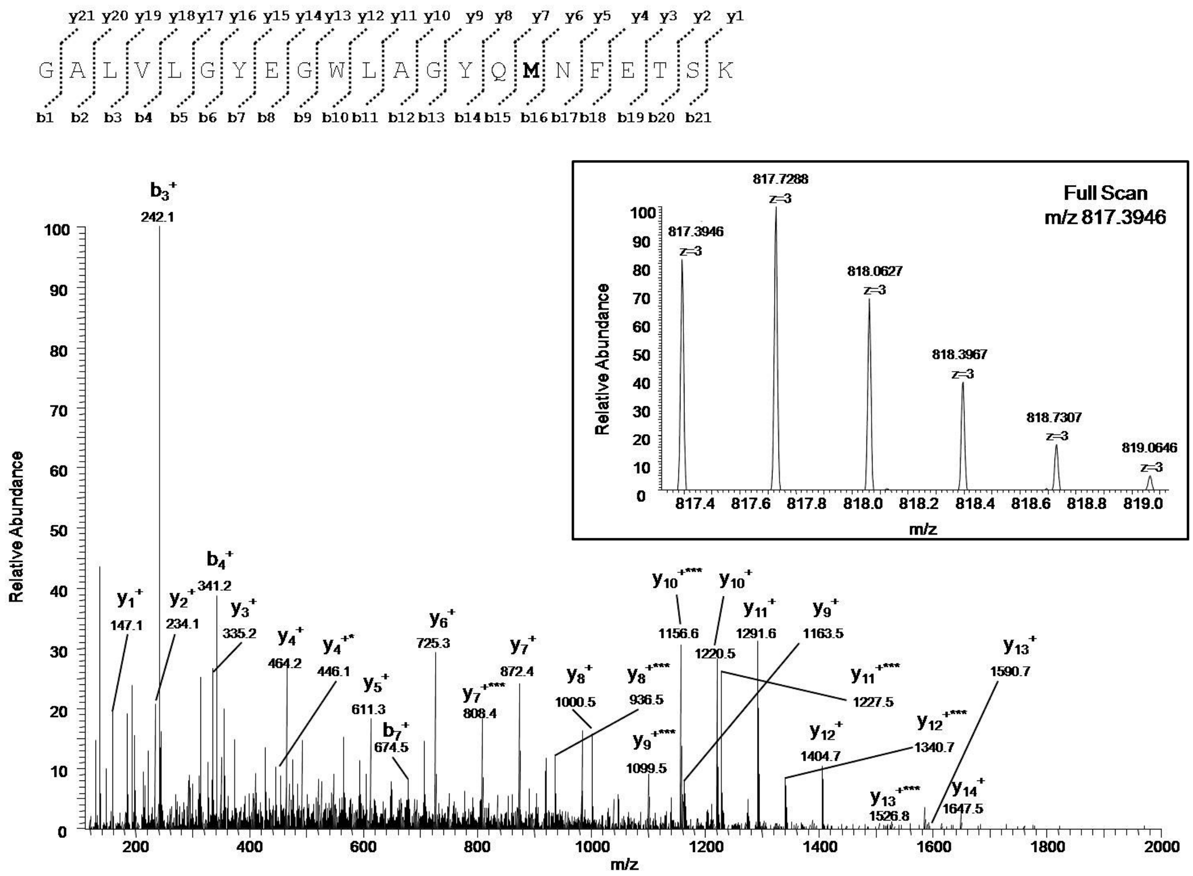

Figure S8. MS/MS spectrum of the triply charged molecular ion at m/z 817.3946 (calculated 817.3951) of the VDAC1 tryptic peptide from NSC34-SOD1G93A cell line containing methionine residue 155 in the oxidized form of methionine sulfoxide. The inset shows the full scan mass spectrum of molecular ion. Fragment ion originated from the neutral loss of H_2O is indicated by an asterisk. Fragment ions originated from the neutral loss of methanesulfenic acid (CH_3SOH, 64 Da) are indicated by three asterisks.

G140 A L V L G Y E G W L A G Y Q **M** N F E T S K161

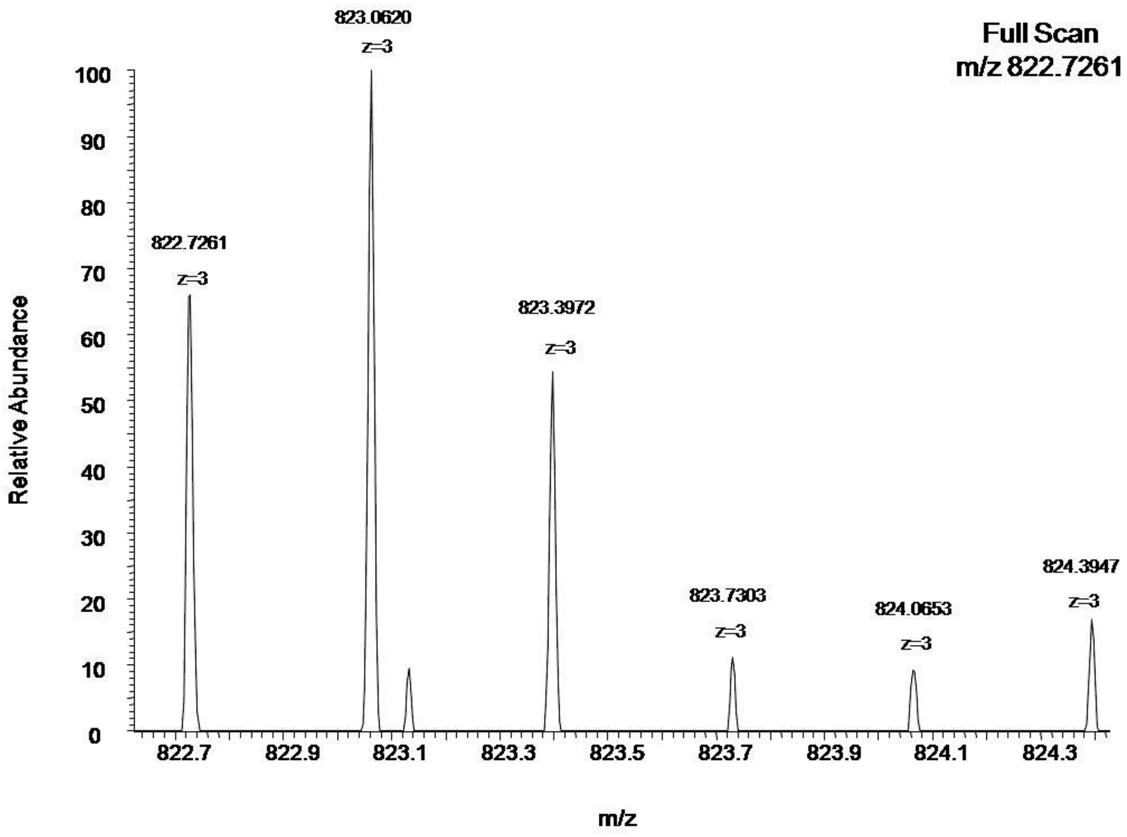

Figure S9. Full scan mass spectrum of the triply charged molecular ion at m/z 822.7261 (calculated 822.7268) of the VDAC1 tryptic peptide from NSC34-SOD1G93A cell line containing methionine residue 155 in the oxidized form of methionine sulfone.

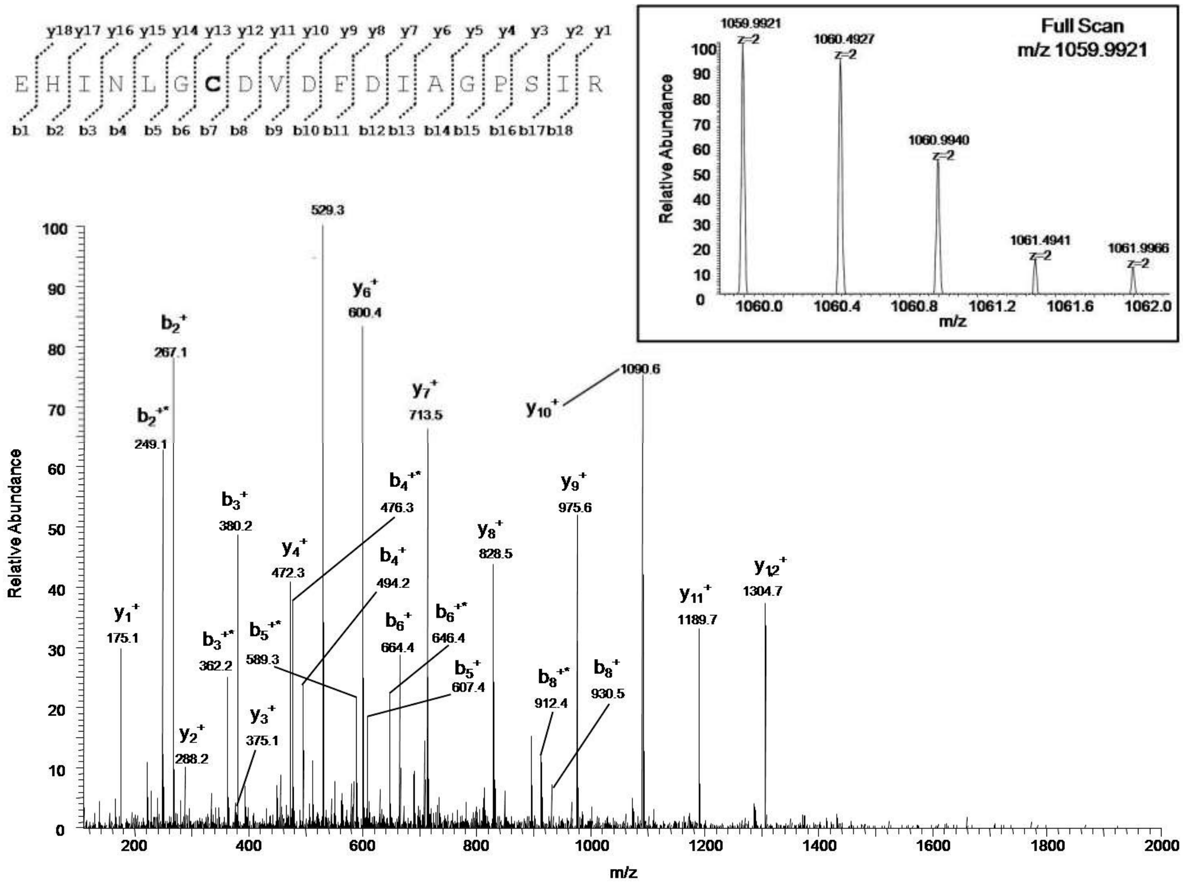

Figure S10. MS/MS spectrum of the doubly charged molecular ion at m/z 1059.9921(calculated 1059.9919) of the VDAC1 tryptic peptide from NSC34-SOD1G93A cell line containing cysteine residue 127 in the form of sulfonic acid. The inset shows the full scan mass spectrum of molecular ion. Fragment ions originated from the neutral loss of H_2O are indicated by an asterisk.

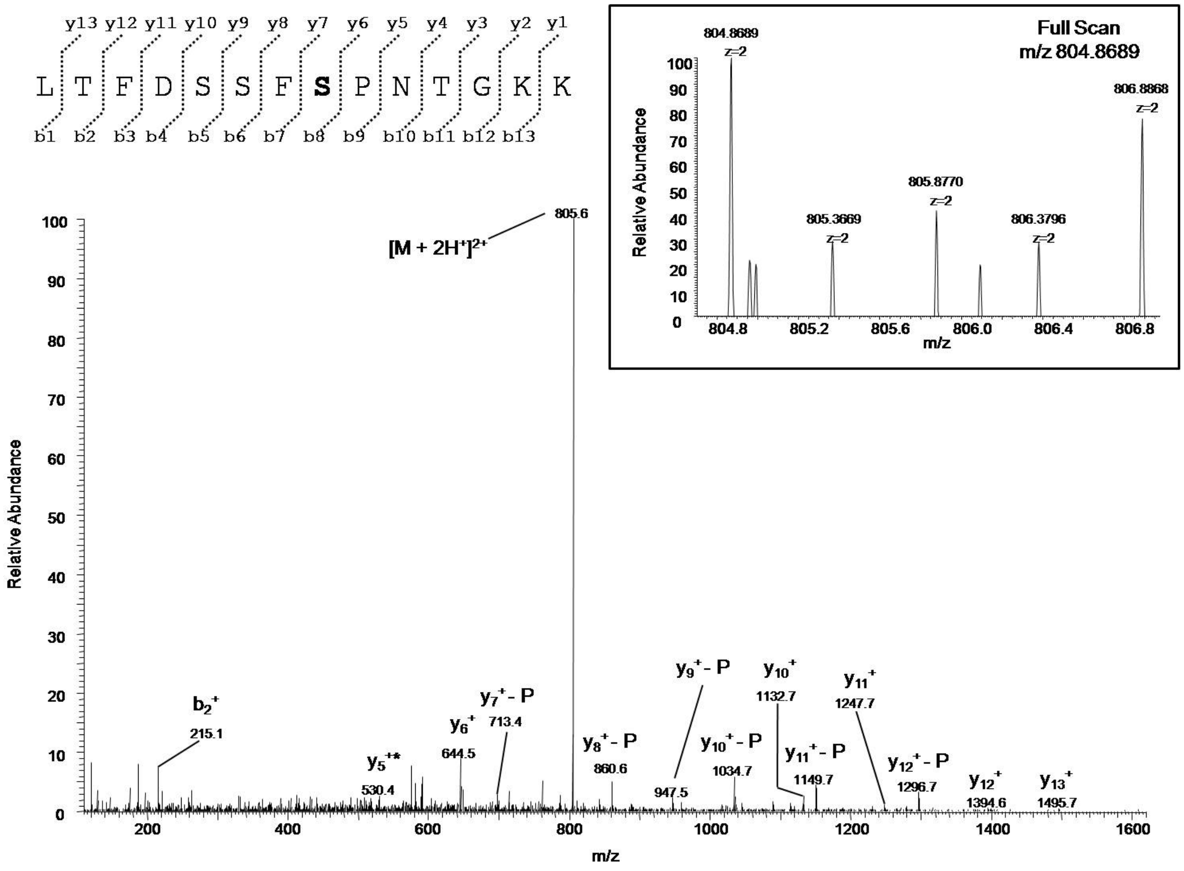

Figure S11. MS/MS spectrum of the doubly charged molecular ion at m/z 804.8689 (calculated 804.8692) of the VDAC1 tryptic peptide from NSC34 cell line containing the serine residue 104 in the phosphorylated form. The inset shows the full scan mass spectrum of molecular ion. Fragment ion originated from the neutral loss of H_2O is indicated by an asterisk.

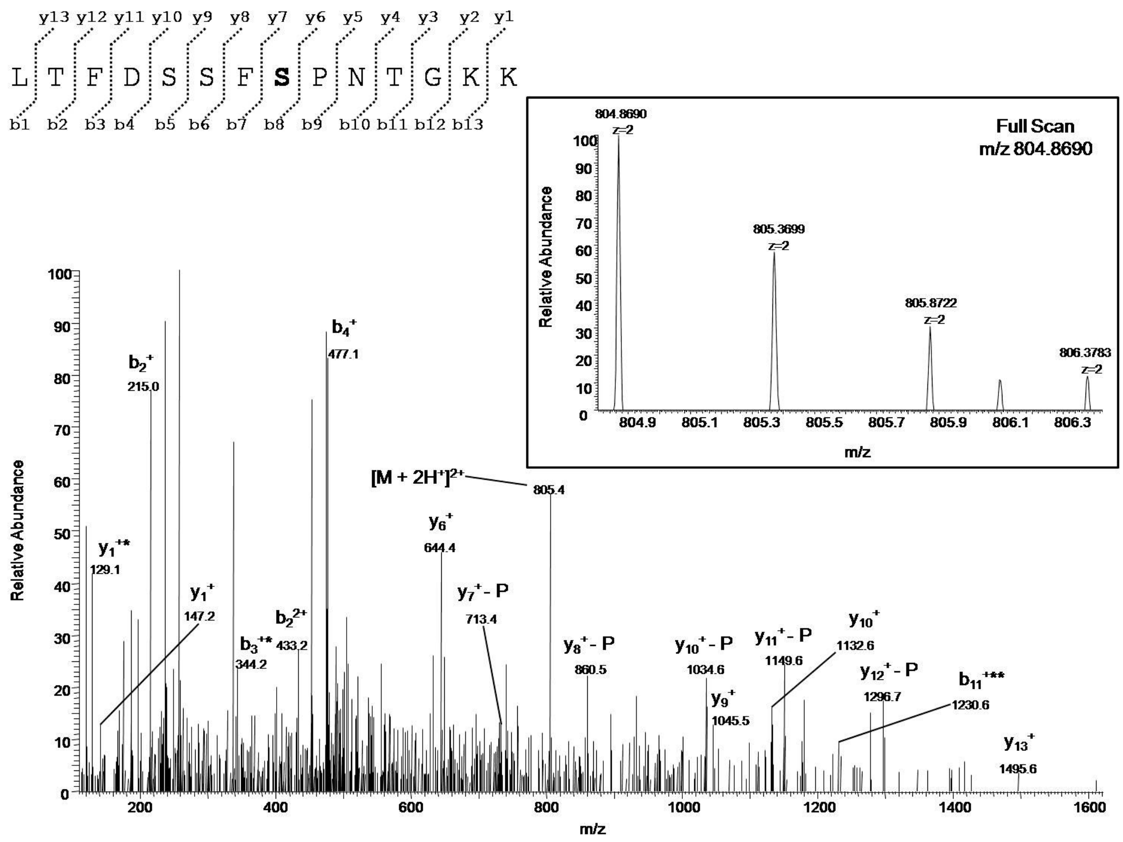

Figure S12. MS/MS spectrum of the doubly charged molecular ion at m/z 804.8690 (calculated 804.8692) of the VDAC1 tryptic peptide from NSC34-SOD1WT cell line containing the serine residue 104 in the phosphorylated form. The inset shows the full scan mass spectrum of molecular ion. Fragment ions originated from the neutral loss of H_2O are indicated by an asterisk. Fragment ion originated from the neutral loss of NH_3 is indicated by two asterisk.

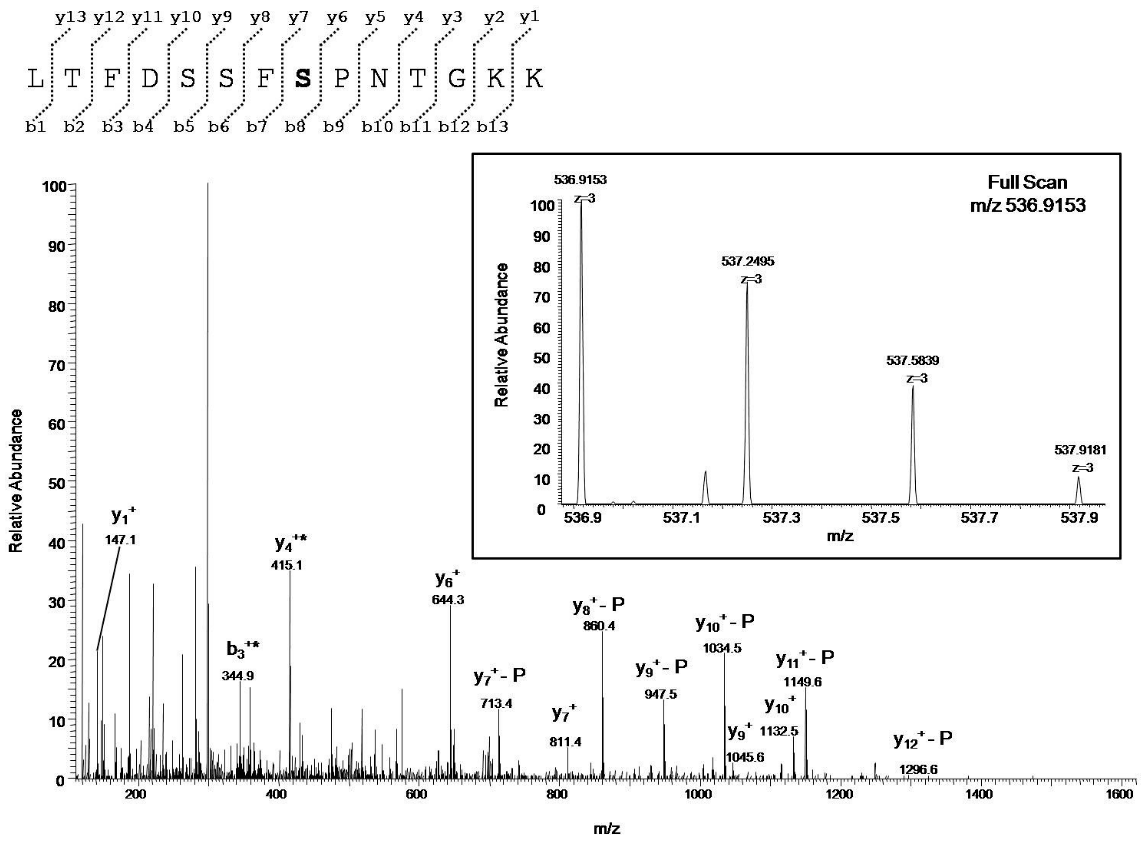

Figure S13. MS/MS spectrum of the triply charged molecular ion at m/z 536.9153 (calculated 536.9154) of the VDAC1 tryptic peptide from NSC34-SOD1G93A cell line containing the serine residue 104 in the phosphorylated form. The inset shows the full scan mass spectrum of molecular ion. Fragment ions originated from the neutral loss of H_2O are indicated by an asterisk.

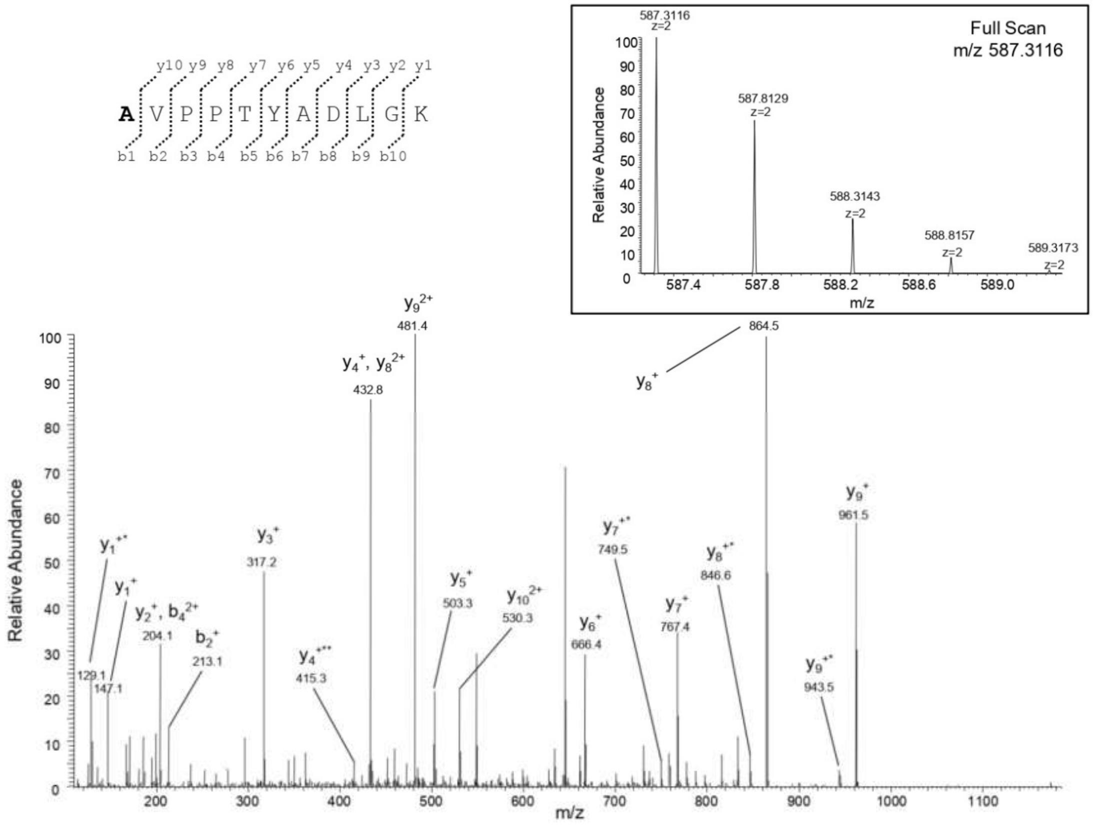

Figure S14. MS/MS spectrum of the doubly charged molecular ion at m/z 587.3116 (calculated 587.3111) of the N-terminal acetylated tryptic peptide of VDAC1 from NSC34 cell line. The inset shows the full scan mass spectrum of molecular ion. Fragment ions originated from the neutral loss of H_2O are indicated by an asterisk. Fragment ion originated from the neutral loss of NH_3 is indicated by two asterisks.

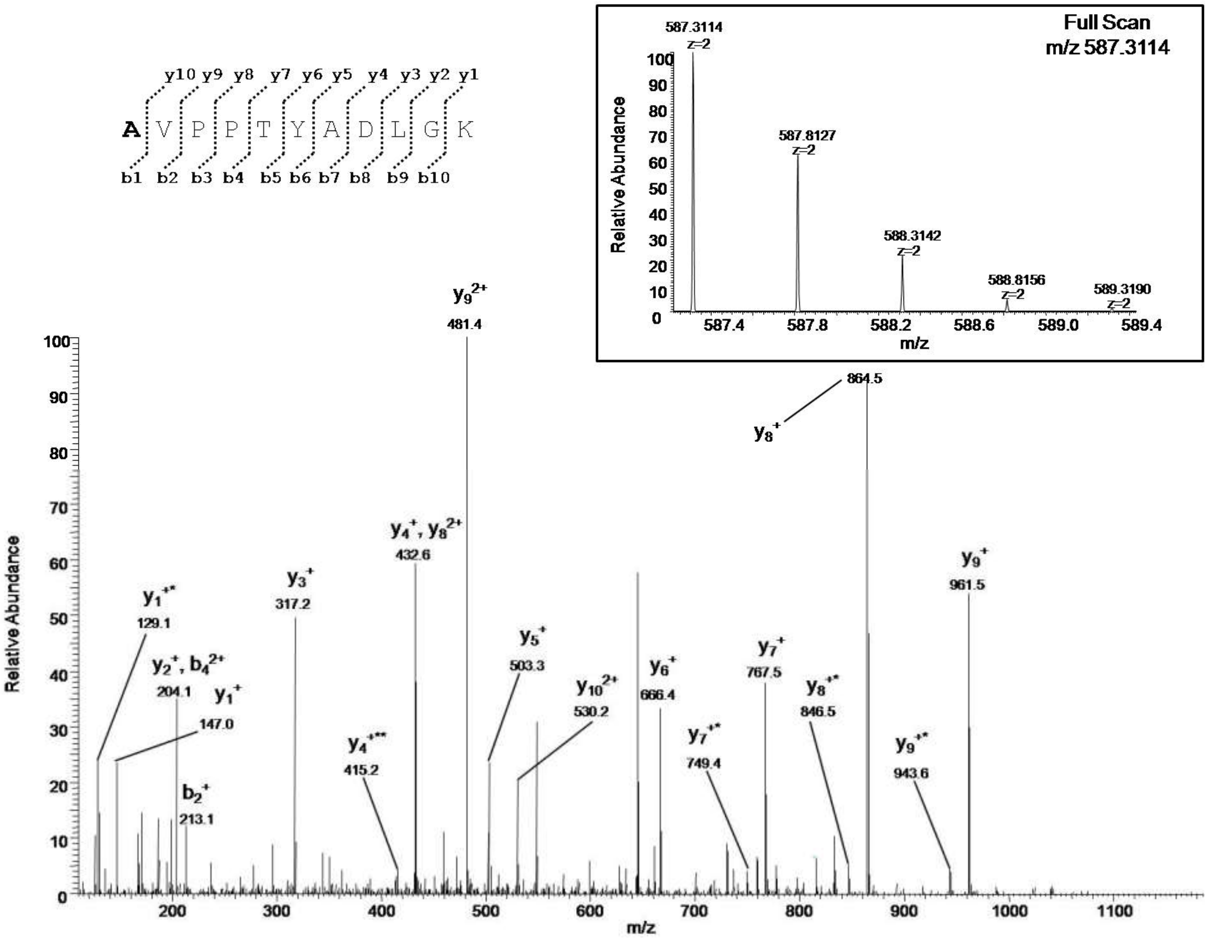

Figure S15. MS/MS spectrum of the doubly charged molecular ion at m/z 587.3114 (calculated 587.3111) of the N-terminal acetylated tryptic peptide of VDAC1 from NSC34-SOD1WT cell line. The inset shows the full scan mass spectrum of molecular ion. Fragment ions originated from the neutral loss of H_2O are indicated by an asterisk. Fragment ion originated from the neutral loss of NH_3 is indicated by two asterisks.

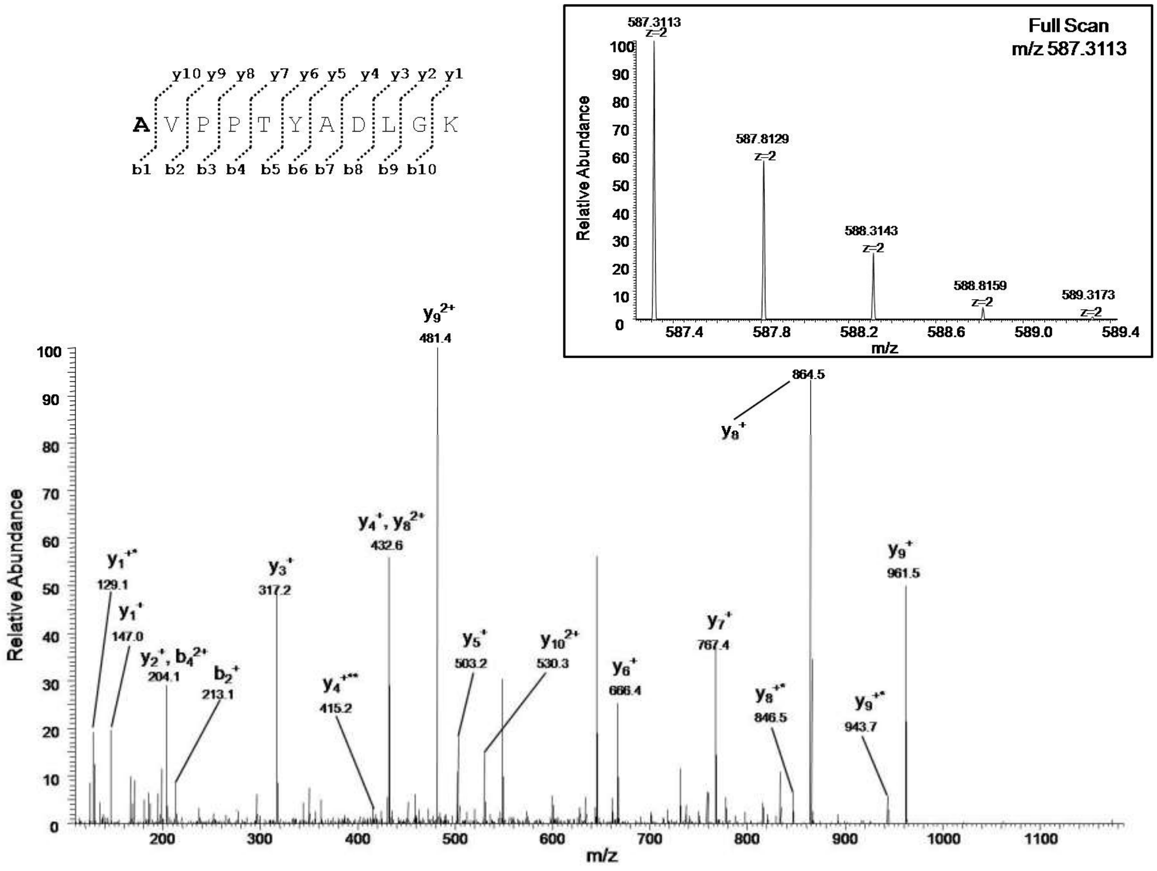

Figure S16. MS/MS spectrum of the doubly charged molecular ion at m/z 587.3113 (calculated 587.3111) of the N-terminal acetylated tryptic peptide of VDAC1 from NSC34-SOD1G93A cell line. The inset shows the full scan mass spectrum of molecular ion. Fragment ions originated from the neutral loss of H_2O are indicated by an asterisk. Fragment ion originated from the neutral loss of NH_3 is indicated by two asterisks.

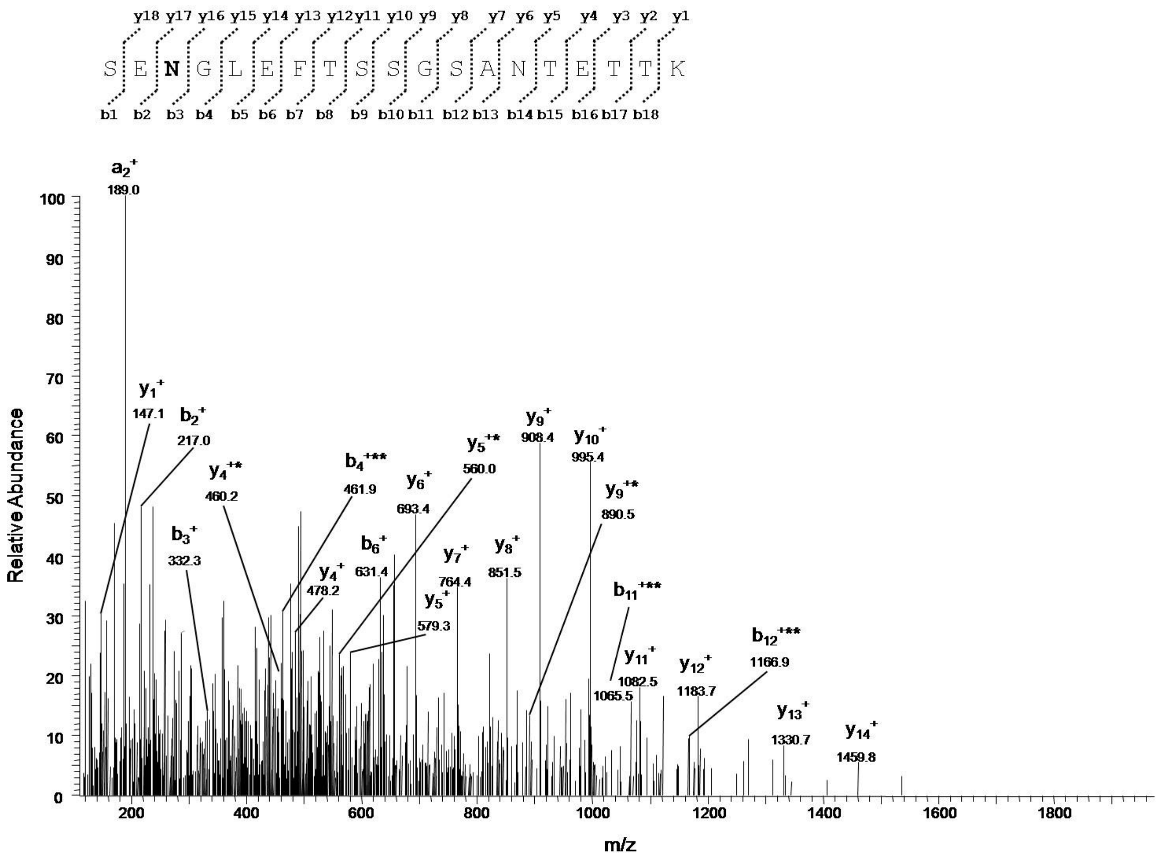

Figure S17. MS/MS spectrum of the triply charged molecular ion at m/z 654.2921 (calculated 654.2925) of the VDAC1 tryptic peptide from NSC34-SOD1G93A cell line containing the asparagine residue 37 in the deamidated form. Fragment ions originated from the neutral loss of H_2O are indicated by an asterisk. Fragment ions originated from the neutral loss of NH_3 are indicated by two asterisk.

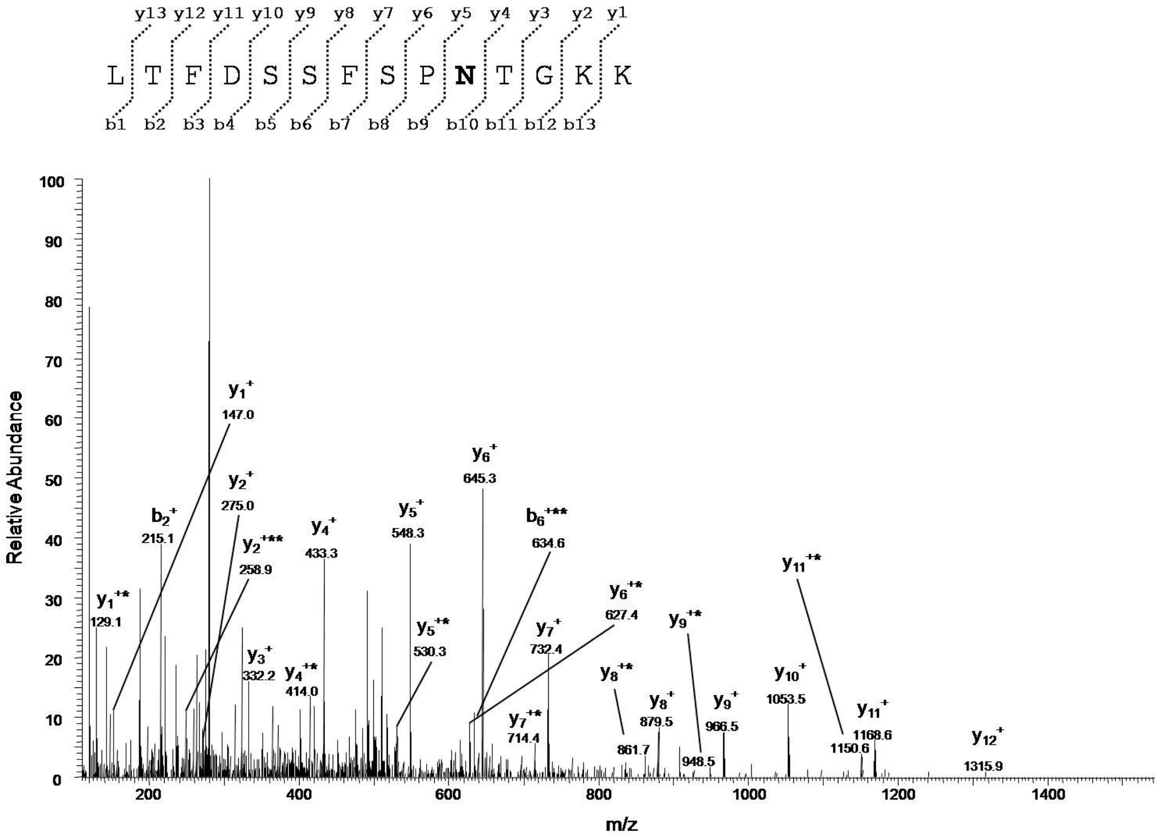

Figure S18. MS/MS spectrum of the triply charged molecular ion at m/z 510.5876 (calculated 510.5880) of the VDAC1 tryptic peptide from NSC34-SOD1G93A cell line containing the asparagine residue 106 in the deamidated form. Fragment ions originated from the neutral loss of H_2O are indicated by an asterisk. Fragment ions originated from the neutral loss of NH_3 are indicated by two asterisk.

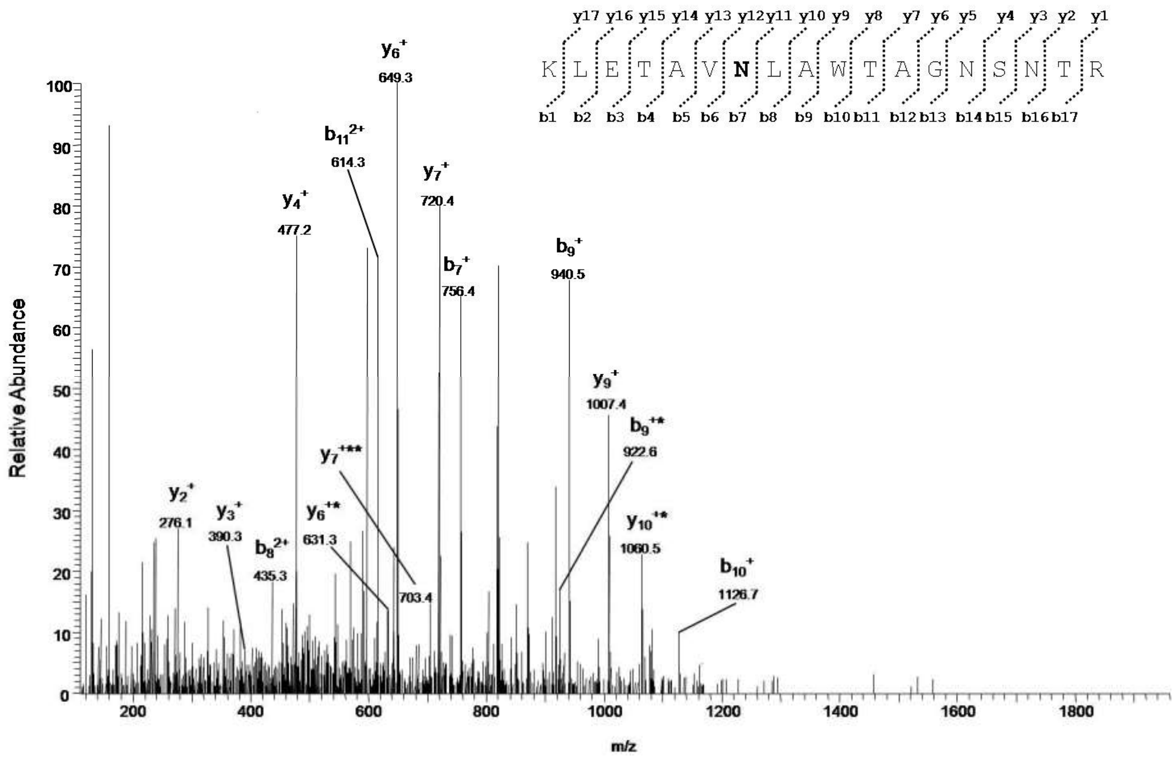

Figure S19. MS/MS spectrum of the triply charged molecular ion at m/z 649.6692 (calculated 649.6696) of the VDAC1 tryptic peptide from NSC34-SOD1G93A cell line containing the asparagine residue 207 in the deamidated form. Fragment ions originated from the neutral loss of H_2O are indicated by an asterisk. Fragment ion originated from the neutral loss of NH_3 is indicated by two asterisk.

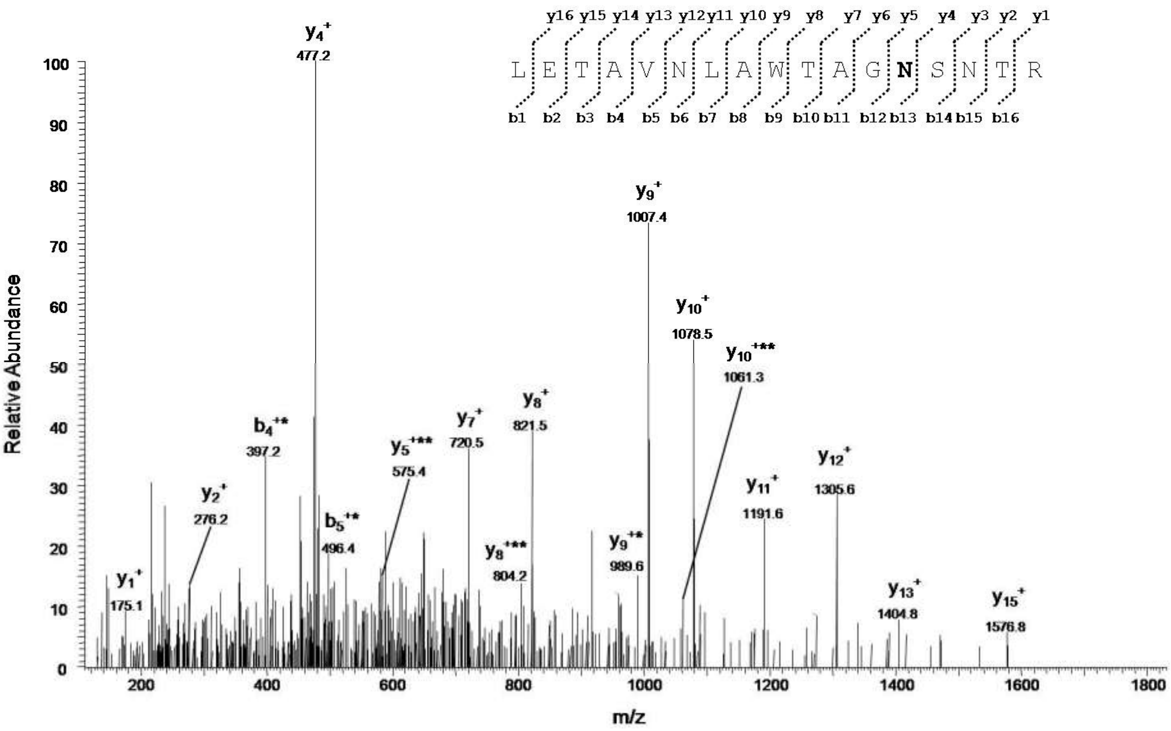

Figure S20. MS/MS spectrum of the doubly charged molecular ion at m/z 909.9527 (calculated 909.9530) of the VDAC1 tryptic peptide from NSC34-SOD1G93A cell line containing the asparagine residue 214 in the deamidated form. Fragment ions originated from the neutral loss of H_2O are indicated by an asterisk. Fragment ions originated from the neutral loss of NH_3 are indicated by two asterisk.

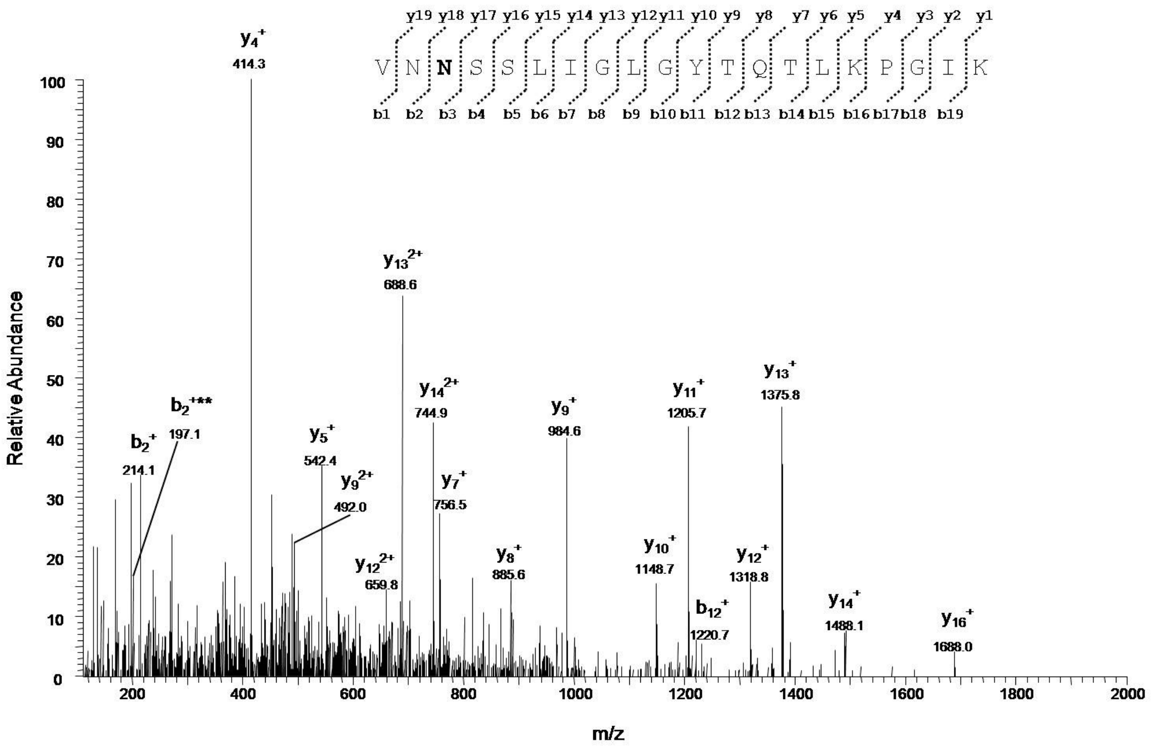

Figure S21. MS/MS spectrum of the triply charged molecular ion at m/z 702.0598 (calculated 702.0602) of the VDAC1 tryptic peptide from NSC34-SOD1G93A cell line containing the asparagine residue 239 in the deamidated form. Fragment ion originated from the neutral loss of NH_3 is indicated by two asterisk.

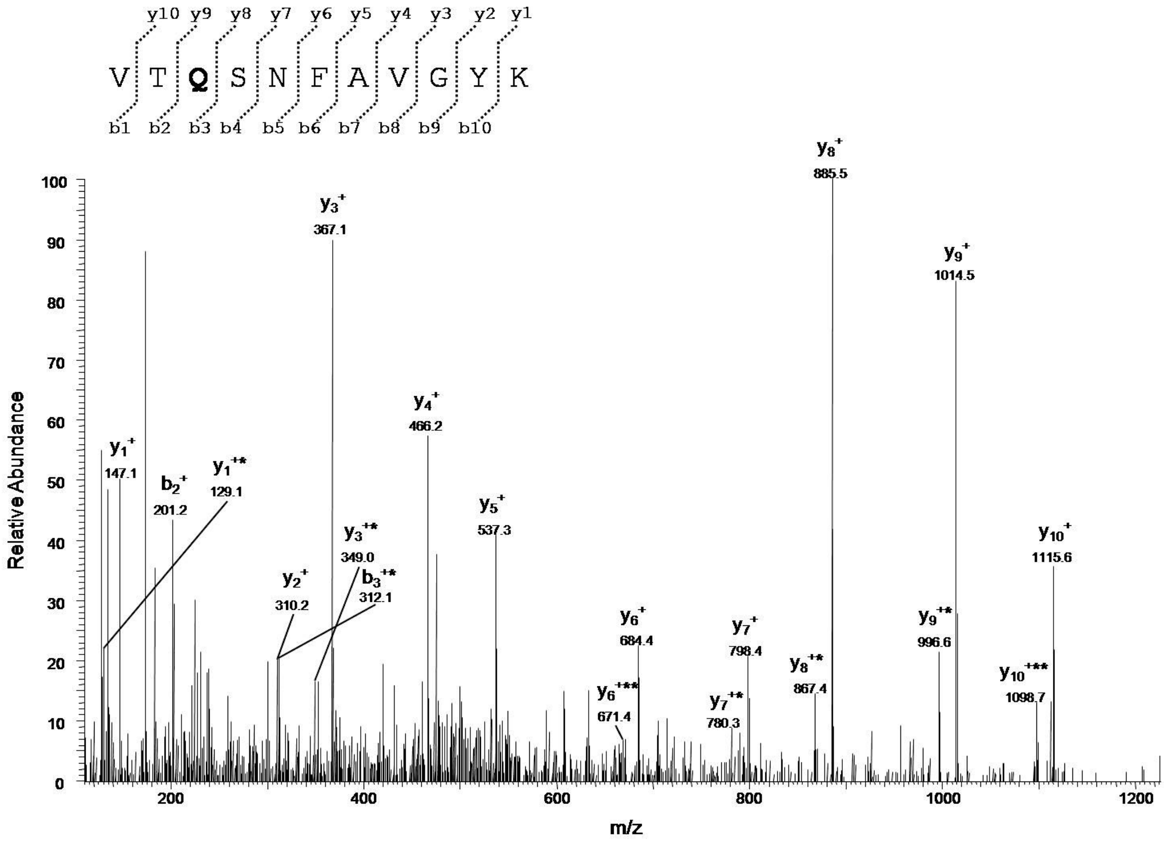

Figure S22. MS/MS spectrum of the doubly charged molecular ion at m/z 607.8062 (calculated 607.8065) of the VDAC1 tryptic peptide from NSC34-SOD1G93A cell line containing the glutamine residue 166 in the deamidated form. Fragment ions originated from the neutral loss of H_2O are indicated by an asterisk. Fragment ions originated from the neutral loss of NH_3 are indicated by two asterisk.

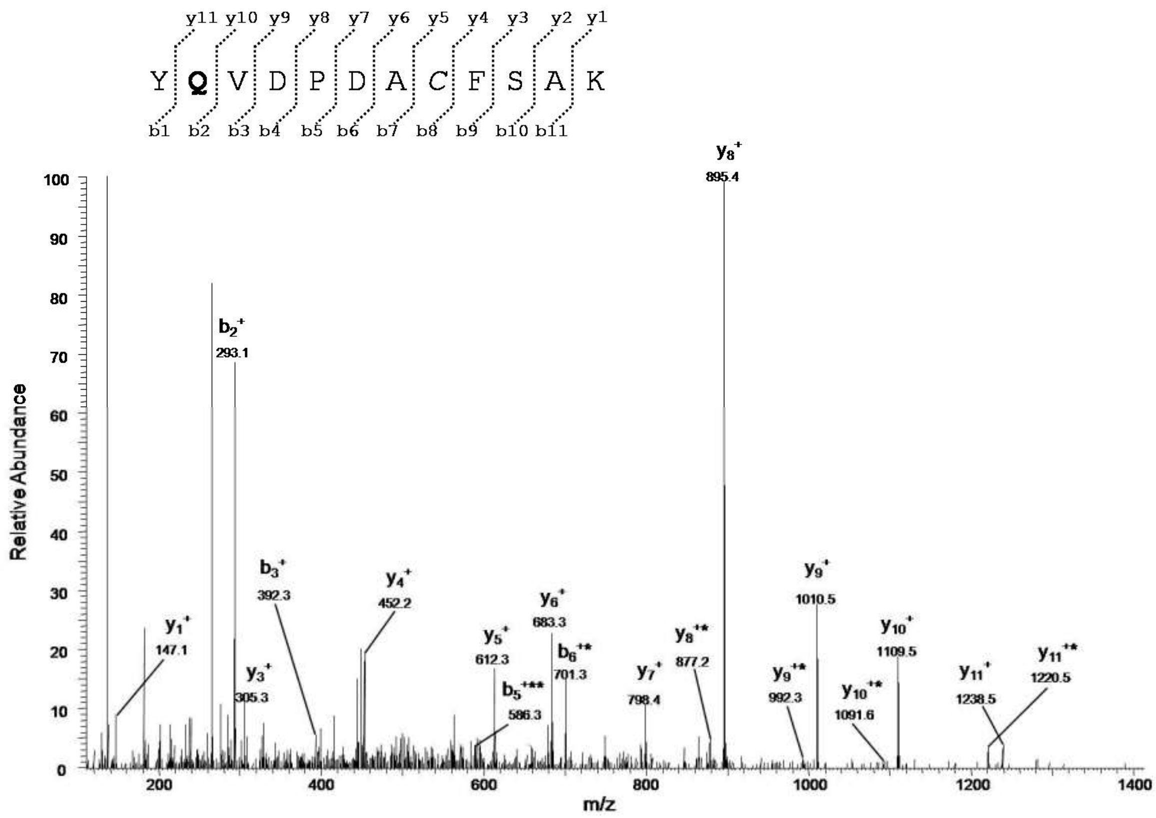

Figure S23. MS/MS spectrum of the doubly charged molecular ion at m/z 701.3032 (calculated 701.3035) of the VDAC1 tryptic peptide from Mouse Motor Neuron-like NSC34-SOD1G93A cell line containing Cys[232] residue in the carboxyamidomethylated form and the glutamine residue 226 in the deamidated form. Fragment ions originated from the neutral loss of H_2O are indicated by an asterisk. Fragment ion originated from the neutral loss of NH_3 is indicated by two asterisk.

SUPPLEMENTARY TABLES

Technical replicate	Frag. n.	Rt (min)	Monoisotopic m/z		Position in the sequence	Peptide sequence
			Measured	Calculated		
I	1	56.41	587.3115 (+2)	587.3111	2-12	*AVPPTYADLGK
II	1	56.25	587.3116 (+2)	587.3111	2-12	*AVPPTYADLGK
III	1	55.70	587.3113 (+2)	587.3111	2-12	*AVPPTYADLGK
I	2	74.10	854.4784 (+1)	854.4771	21-28	GYGFGLIK
II	2	74.73	427.7423 (+2)	427.7422	21-28	GYGFGLIK
III	2	68.00	854.4779 (+1)	854.4771	21-28	GYGFGLIK
I	3	70.48	730.3455 (+3)	730.3450	33-53	TKSENGLEFTSSGSANTETTK
II	3	69.83	730.3453 (+3)	730.3450	33-53	TKSENGLEFTSSGSANTETTK
III	3	65.16	730.3462 (+3)	730.3450	33-53	TKSENGLEFTSSGSANTETTK
I	4	63.55	980.4435 (+2)	980.4425	35-53	SENGLEFTSSGSANTETTK
II	4	63.98	980.4438 (+2)	980.4425	35-53	SENGLEFTSSGSANTETTK
III	4	53.18	980.4437 (+2)	980.4425	35-53	SENGLEFTSSGSANTETTK
I	5	57.64	424.2294 (+2)	424.2296	54-61	VNGSLETK
II	5	57.43	424.2294 (+2)	424.2296	54-61	VNGSLETK
I	6	73.28	687.8329 (+2)	687.8324	64-74	WTEYGLTFTEK
II	6	73.68	687.8329 (+2)	687.8324	64-74	WTEYGLTFTEK
III	6	67.40	687.8334 (+2)	687.8324	64-74	WTEYGLTFTEK
I	7	71.19	1088.5300 (+2)	1088.5295	75-93	WNTDNTLGTEITVEDQLAR
II	7	71.35	1088.5299 (+2)	1088.5295	75-93	WNTDNTLGTEITVEDQLAR
III	7	66.81	1088.5298 (+2)	1088.5295	75-93	WNTDNTLGTEITVEDQLAR
I	8	66.59	700.8389 (+2)	700.8383	97-109	LTFDSSFSPNTGK
II	8	68.31	700.8389 (+2)	700.8383	97-109	LTFDSSFSPNTGK
III	8	59.62	700.8389 (+2)	700.8383	97-109	LTFDSSFSPNTGK
I	9	73.92	764.8861 (+2)	764.8857	97-110	LTFDSSFSPNTGKK
II	9	74.56	510.2605 (+3)	510.2596	97-110	LTFDSSFSPNTGKK
III	9	67.58	764.8867 (+2)	764.8857	97-110	LTFDSSFSPNTGKK
I	10	76.12	1217.5920 (+2)	1217.5910	140-161	GALVLGYEGWLAGYQMNFETSK

II	10	77.19	1217.5913 (+2)	1217.5910	140-161	GALVLGYEGWLAGYQMNFETSK
III	10	72.16	1217.5920 (+2)	1217.5910	140-161	GALVLGYEGWLAGYQMNFETSK
I	11	65.85	607.3149 (+2)	607.3142	164-174	VTQSNFAVGYK
II	11	67.61	607.3151 (+2)	607.3142	164-174	VTQSNFAVGYK
III	11	55.69	607.3145 (+2)	607.3142	164-174	VTQSNFAVGYK
I	12	73.77	867.4022 (+3)	867.4015	175-197	TDEFQLHTNVNDGTEFGGSIYQK
II	12	74.39	867.4025 (+3)	867.4015	175-197	TDEFQLHTNVNDGTEFGGSIYQK
III	12	67.53	867.4031 (+3)	867.4015	175-197	TDEFQLHTNVNDGTEFGGSIYQK
I	13	74.19	973.5087 (+2)	973.5081	201-218	KLETAVNLAWTAGNSNTR
II	13	74.91	649.3420 (+3)	649.3412	201-218	KLETAVNLAWTAGNSNTR
III	13	68.20	973.5085 (+2)	973.5081	201-218	KLETAVNLAWTAGNSNTR
I	14	67.45	606.6436 (+3)	606.6429	202-218	LETAVNLAWTAGNSNTR
II	14	68.63	909.4628 (+2)	909.4607	202-218	LETAVNLAWTAGNSNTR
III	14	64.12	909.4619 (+2)	909.4607	202-218	LETAVNLAWTAGNSNTR
I	15	65.85	700.8120 (+2)	700.8112	225-236	YQVDPDA***C***FSAK
II	15	67.52	700.8120 (+2)	700.8112	225-236	YQVDPDA***C***FSAK
III	15	55.99	700.8119 (+2)	700.8112	225-236	YQVDPDA***C***FSAK
I	16	74.28	701.7324 (+3)	701.7318	237-256	VNNSSLIGLGYTQTLKPGIK
II	16	74.99	1052.0951 (+2)	1052.0941	237-256	VNNSSLIGLGYTQTLKPGIK
III	16	68.45	1052.0952 (+2)	1052.0941	237-256	VNNSSLIGLGYTQTLKPGIK

*A: N-terminal acetylated; ***C***: cysteine carboxyamidomethylated.

Table S1. Tryptic peptides found in the analysis of VDAC1 from NSC34 cell line after DTT reduction and carboxyamidomethylation.

Retention time, experimentally measured and calculated monoisotopic m/z of the molecular ions, position in the sequence and peptide sequence of fragments present in the tryptic digest analyzed in triplicateof reduced and carboxyamidomethylated VDAC1 are reported. All sequences were confirmed by MS/MS. These sequences were used to build the sequence coverage reported in Figure 1.

Technical replicate	Frag. n.	Rt (min)	Monoisotopic m/z		Position in the sequence	Peptide sequence
			Measured	Calculated		
I	1	69.80	744.3972 (+2)	744.3968	2-15	*AVPPTYADLGKSAR
I	2	74.92	924.9792 (+2)	924.9782	2-18	*AVPPTYADLGKSARDVF
II	2	79.08	924.9794 (+2)	924.9782	2-18	*AVPPTYADLGKSARDVF
III	2	70.37	924.9790 (+2)	924.9782	2-18	*AVPPTYADLGKSARDVF
I	3	76.20	589.8124 (+2)	589.8118	8-18	ADLGKSARDVF
II	4	80.35	554.2932 (+2)	554.2938	9-18	DLGKSARDVF
II	5	80.51	440.2375 (+2)	440.2383	11-18	GKSARDVF
I	6	75.85	690.8544 (+2)	690.8545	30-41	DLKTKSENGLEF
II	6	80.24	690.8545 (+2)	690.8545	30-41	DLKTKSENGLEF
III	6	70.30	690.8544 (+2)	690.8545	30-41	DLKTKSENGLEF
I	7	72.26	725.6863 (+3)	725.6852	42-62	TSSGSANTETTKVNGSLETKY
II	7	78.20	1088.0245 (+2)	1088.0248	42-62	TSSGSANTETTKVNGSLETKY
III	7	66.57	725.6863 (+3)	725.6852	42-62	TSSGSANTETTKVNGSLETKY
I	8	69.82	995.4913 (+2)	995.4904	76-93	NTDNTLGTEITVEDQLAR
II	8	78.01	995.4908 (+2)	995.4904	76-93	NTDNTLGTEITVEDQLAR
III	8	62.60	995.4905 (+2)	995.4904	76-93	NTDNTLGTEITVEDQLAR
I	9	74.75	1080.5437 (+2)	1080.5431	76-95	NTDNTLGTEITVEDQLARGL
II	9	78.40	720.6985 (+3)	720.6980	76-95	NTDNTLGTEITVEDQLARGL
III	9	70.51	720.7000 (+3)	720.6980	76-95	NTDNTLGTEITVEDQLARGL
I	10	69.62	666.3447 (+2)	666.3443	82-93	GTEITVEDQLAR
III	10	58.98	666.3445 (+2)	666.3443	82-93	GTEITVEDQLAR
III	11	68.90	751.3962 (+2)	751.3970	82-95	GTEITVEDQLARGL
II	12	80.85	681.6891 (+3)	681.6889	100-118	DSSFSPNTGKKNAKIKTGY
I	13	77.16	536.3014 (+3)	536.3024	104-118	SPNTGKKNAKIKTGY
II	13	81.14	536.3018 (3)	536.3025	104-118	SPNTGKKNAKIKTGY
I	14	77.29	436.9257 (+3)	436.9265	107-118	TGKKNAKIKTGY
II	14	81.30	436.9257 (+3)	436.9265	107-118	TGKKNAKIKTGY
II	15	80.19	447.7562 (+2)	447.7564	111-118	NAKIKTGY
I	16	70.15	535.3041 (+2)	535.3042	132-142	DIAGPSIRGAL
II	16	78.30	535.3036 (+2)	535.3042	132-142	DIAGPSIRGAL

III	16	63.74	535.3042 (+2)	535.3042	132-142	DIAGPSIRGAL
II	17	70.82	823.3993 (+1)	823.3990	143-149	VLGYEGW
II	18	79.72	648.8313 (+2)	648.8313	156-166	NFETSKSRVTQ
I	19	75.85	822.9034 (+2)	822.9030	156-169	NFETSKSRVTQSNF
II	19	80.24	822.9035 (+2)	822.9030	156-169	NFETSKSRVTQSNF
III	19	70.13	822.9040 (+2)	822.9030	156-169	NFETSKSRVTQSNF
I	20	74.77	692.3475 (+2)	692.3473	158-169	ETSKSRVTQSNF
II	20	79.72	692.3474 (+2)	692.3473	158-169	ETSKSRVTQSNF
III	21	54.98	679.3050 (+2)	679.3051	179-190	**Q**LHTNVNDGTEF
I	22	69.73	687.8185 (+2)	687.8184	179-190	QLHTNVNDGTEF
II	22	78.16	687.8188 (+2)	687.8184	179-190	QLHTNVNDGTEF
III	22	59.62	687.8186 (+2)	687.8184	179-190	QLHTNVNDGTEF
II	23	61.49	917.9168 (+2)	917.9163	179-195	**Q**LHTNVNDGTEFGGSIY
III	23	61.03	917.9177 (+2)	917.9163	179-195	**Q**LHTNVNDGTEFGGSIY
II	24	78.20	926.4301 (+2)	926.4296	179-195	QLHTNVNDGTEFGGSIY
III	25	33.87	567.2468 (+2)	567.2471	181-190	HTNVNDGTEF
I	26	75.91	734.4307 (+2)	734.4306	196-208	**Q**KVNKKLETAVNL
II	26	80.35	734.4309 (+2)	734.4306	196-208	**Q**KVNKKLETAVNL
III	26	70.32	489.9568 (+3)	489.9564	196-208	**Q**KVNKKLETAVNL
I	27	76.96	742.9445 (+2)	742.9439	196-208	QKVNKKLETAVNL
II	27	80.97	742.9441 (+2)	742.9439	196-208	QKVNKKLETAVNL
II	28	78.20	612.7917 (+2)	612.7919	209-219	AWTAGNSNTRF
III	28	61.20	612.7915 (+2)	612.7919	209-219	AWTAGNSNTRF
I	29	69.65	484.2335 (+2)	484.2338	211-219	TAGNSNTRF
III	29	57.47	484.2336 (+2)	484.2338	211-219	TAGNSNTRF
I	30	48.42	476.1978 (+2)	476.1980	226-233	QVDPDA***C***F
II	30	48.86	476.1978 (+2)	476.1980	226-233	QVDPDA***C***F
III	30	48.79	476.1974 (+2)	476.1980	226-233	QVDPDA***C***F
I	31	72.52	601.8415 (+2)	601.8412	234-245	SAKVNNSSLIGL
II	31	78.35	601.8408 (+2)	601.8412	234-245	SAKVNNSSLIGL
III	31	67.69	601.8412 (+2)	601.8412	234-245	SAKVNNSSLIGL
I	32	72.27	711.8839 (+2)	711.8835	234-247	SAKVNNSSLIGLGY
II	32	78.30	711.8836 (+2)	711.8835	234-247	SAKVNNSSLIGLGY
III	32	67.64	711.8840 (+2)	711.8835	234-247	SAKVNNSSLIGLGY

I	33	76.13	549.8478 (+2)	549.8482	248-257	TQTLKPGIKL
II	33	80.51	549.8481 (+2)	549.8482	248-257	TQTLKPGIKL
III	34	70.58	549.8477 (+2)	549.8482	248-257	TQTLKPGIKL
I	35	76.76	531.9620 (+3)	531.9623	260-275	SALLDGKNVNAGGHKL
II	35	80.80	531.9617 (+3)	531.9623	260-275	SALLDGKNVNAGGHKL
I	36	76.15	403.8825 (+3)	403.8832	264-275	DGKNVNAGGHKL
II	36	80.42	605.3212 (+2)	605.3209	264-275	DGKNVNAGGHKL
III	36	70.51	403.8829 (+3)	403.8832	264-275	DGKNVNAGGHKL

*A: N-terminal acetylated; ***C***: cysteine carboxyamidomethylated; **Q**: pyroglutamic acid form.

Table S2. Chymotryptic peptides found in the analysis of VDAC1 from NSC34 cell line after DTT reduction and carboxyamidomethylation.

Retention time, experimentally measured and calculated monoisotopic m/z of the molecular ions, position in the sequence and peptide sequence of fragments present in the chymotryptic digest analyzed in triplicate of reduced and carboxyamidomethylated VDAC1 are reported. All sequences were confirmed by MS/MS. These sequences were used to build the sequence coverage reported in Figure 1.

Frag. n.	Technical replicate	Rt (min)	Monoisotopic m/z		Position in the sequence	Peptide sequence
			Measured	Calculated		
1	I	71.93	1059.9926 (+2)	1059.9919	121-139	EHINLG**C**DVDFDIAGPSIR
	II	72.12	1059.9929 (+2)			
	III	67.55	1059.9923 (+2)			
2	I	75.46	1225.5891 (+2)	1225.5888	140-161	GALVLGYEGWLAGYQ**M**NFETSK
	II	76.47	817.3961 (+3)	817.3951		
	III	70.85	817.3960 (+3)			
3	I	74.63	1233.5824 (+2)	1233.5862	140-161	GALVLGYEGWLAGYQ**M**NFETSK
	II	74.99	1233.5898 (+2)			
	III	69.20	1233.5890 (+2)			

C: cysteine oxidized to sulfonic acid; **M**: methionine sulfoxide; **M**: methionine sulfone.

Table S3. Retention time, experimentally measured and calculated monoisotopic m/z of the molecular ions, position in the sequence and peptide sequence of sulfur containing tryptic fragments found in the analysis of VDAC1 from NSC34 cell line digest reduced with DTT, carboxyamidomethylated and digested in-solution. Fragment 1 was used to build the sequence coverage reported in Figure 1.

Frag. n.	Technical replicate	Rt (min)	Monoisotopic m/z		Position in the sequence	Peptide sequence	MS/MS
			Measured	Calculated			
1	I	76.36	531.9160 (+3)	531.9161	119-131	KREHINLG**C**DVDF	Yes
	II	79.62	531.9169 (+3)	531.9161			No
	III	70.80	531.9161 (+3)	531.9161			No

C: cysteine oxidized to sulfonic acid.

Table S4. Retention time, experimentally measured and calculated monoisotopic m/z of the molecular ion of the chymotryptic peptide K^{119}REHINLG**C**DVDF^{131} found in VDAC1 from NSC34 cell line digest reduced with DTT, carboxyamidomethylated and digested in-solution. This fragment was used to build the sequence coverage reported in Figure 1.

Technical replicate	Peptide	Measured monoisotopic *m/z*	Absolute intensity	Ratio Ox/Red
I	GALVLGYEGWLAGYQ**M**NFETSK	1225.5891 (+2)	$5.1 \cdot 10^{6}$	5.6
I	GALVLGYEGWLAGYQMNFETSK	1217.5920 (+2)	$9.1 \cdot 10^{5}$	
II	GALVLGYEGWLAGYQ**M**NFETSK	817.3961 (+3)	$8.1 \cdot 10^{6}$	3.5
II	GALVLGYEGWLAGYQMNFETSK	1217.5913 (+2)	$2.3 \cdot 10^{6}$	
III	GALVLGYEGWLAGYQ**M**NFETSK	817.3960 (+3)	$1.4 \cdot 10^{6}$	5.0
III	GALVLGYEGWLAGYQMNFETSK	1217.5920 (+2)	$2.8 \cdot 10^{5}$	
I	GALVLGYEGWLAGYQ**M**NFETSK	1233.5824 (+2)	$9.9 \cdot 10^{4}$	0.1
I	GALVLGYEGWLAGYQMNFETSK	1217.5920 (+2)	$9.1 \cdot 10^{5}$	
II	GALVLGYEGWLAGYQ**M**NFETSK	1233.5898 (+2)	$1.6 \cdot 10^{5}$	0.1
II	GALVLGYEGWLAGYQMNFETSK	1217.5913 (+2)	$2.3 \cdot 10^{6}$	
III	GALVLGYEGWLAGYQ**M**NFETSK	1233.5890 (+2)	$5.5 \cdot 10^{4}$	0.2
III	GALVLGYEGWLAGYQMNFETSK	1217.5920 (+2)	$2.8 \cdot 10^{5}$	

M: methionine sulfoxide; **M**: methionine sulfone.

Table S5. Ratio of the absolute intensities of the molecular ions of the sulfur and not sulfur containing tryptic peptide $G^{140}ALVLGYEGWLAGYQMNFETSK^{161}$ found in the analysis of VDAC1 from NSC34 cell line HTP-preparation reduced with DTT, carboxyamidomethylated and digested in-solution.

Technical replicate	Frag. n.	Rt (min)	Monoisotopic m/z		Position in the sequence	Peptide sequence
			Measured	Calculated		
I	1	55.03	587.3114 (+2)	587.3111	2-12	*AVPPTYADLGK
II	1	55.98	1173.6157 (+1)	1173.6150	2-12	*AVPPTYADLGK
III	1	55.08	587.3114 (+2)	587.3111	2-12	*AVPPTYADLGK
I	2	66.88	427.7418 (+2)	427.7422	21-28	GYGFGLIK
II	2	70.11	854.4781 (+1)	854.4771	21-28	GYGFGLIK
III	2	62.81	427.7418 (+2)	427.7422	21-28	GYGFGLIK
I	3	65.77	730.3456 (+3)	730.3450	33-53	TKSENGLEFTSSGSANTETTK
II	3	66.67	730.3463 (+3)	730.3450	33-53	TKSENGLEFTSSGSANTETTK
III	3	61.40	730.3453 (+3)	730.3450	33-53	TKSENGLEFTSSGSANTETTK
I	4	54.05	980.4437 (+2)	980.4425	35-53	SENGLEFTSSGSANTETTK
II	4	56.90	980.4442 (+2)	980.4425	35-53	SENGLEFTSSGSANTETTK
III	4	47.21	980.4431 (+2)	980.4425	35-53	SENGLEFTSSGSANTETTK
II	5	54.46	424.2294 (+2)	424.2296	54-61	VNGSLETK
I	6	68.01	687.8331 (+2)	687.8324	64-74	WTEYGLTFTEK
II	6	69.39	687.8331 (+2)	687.8324	64-74	WTEYGLTFTEK
III	6	63.72	687.8329 (+2)	687.8324	64-74	WTEYGLTFTEK
I	7	67.31	1088.5300 (+2)	1088.5295	75-93	WNTDNTLGTEITVEDQLAR
II	7	68.28	1088.5302 (+2)	1088.5295	75-93	WNTDNTLGTEITVEDQLAR
III	7	63.64	1088.5303 (+2)	1088.5295	75-93	WNTDNTLGTEITVEDQLAR
I	8	59.73	700.8387 (+2)	700.8383	97-109	LTFDSSFSPNTGK
II	8	61.88	700.8389 (+2)	700.8383	97-109	LTFDSSFSPNTGK
III	8	57.52	700.8390 (+2)	700.8383	97-109	LTFDSSFSPNTGK
I	9	68.22	764.8862 (+2)	764.8857	97-110	LTFDSSFSPNTGKK
II	9	70.00	764.8864 (+2)	764.8857	97-110	LTFDSSFSPNTGKK
III	9	63.88	764.8864 (+2)	764.8857	97-110	LTFDSSFSPNTGKK
I	10	72.20	1217.5923 (+2)	1217.5910	140-161	GALVLGYEGWLAGYQMNFETSK
II	10	73.70	1217.5917 (+2)	1217.5910	140-161	GALVLGYEGWLAGYQMNFETSK
III	10	70.88	812.0640 (+3)	812.0631	140-161	GALVLGYEGWLAGYQMNFETSK
I	11	56.05	607.3148 (+2)	607.3142	164-174	VTQSNFAVGYK
II	11	59.06	607.3146 (+2)	607.3142	164-174	VTQSNFAVGYK

III	11	50.30	607.3151 +2)	607.3142	164-174	VTQSNFAVGYK
I	12	68.16	867.4025 (+3)	867.4015	175-197	TDEFQLHTNVNDGTEFGGSIYQK
II	12	69.79	867.4028 (+3)	867.4015	175-197	TDEFQLHTNVNDGTEFGGSIYQK
III	12	64.01	867.4027 (+3)	867.4015	175-197	TDEFQLHTNVNDGTEFGGSIYQK
I	13	68.77	973.5092 (+2)	973.5081	201-218	KLETAVNLAWTAGNSNTR
II	13	70.66	973.5093 (+2)	973.5081	201-218	KLETAVNLAWTAGNSNTR
III	13	65.09	973.5096 (+2)	649.3412	201-218	KLETAVNLAWTAGNSNTR
I	14	64.35	909.4612 (+2)	909.4607	202-218	LETAVNLAWTAGNSNTR
II	14	65.09	909.4620 (+2)	909.4607	202-218	LETAVNLAWTAGNSNTR
III	14	61.65	606.6434 (+3)	909.4607	202-218	LETAVNLAWTAGNSNTR
I	15	56.20	700.8120 (+2)	700.8112	225-236	YQVDPDA***C***FSAK
II	15	58.94	700.8120 (+2)	700.8112	225-236	YQVDPDA***C***FSAK
III	15	51.48	700.8120 (+2)	700.8112	225-236	YQVDPDA***C***FSAK
I	16	68.91	1052.0967 (+2)	1052.0941	237-256	VNNSSLIGLGYTQTLKPGIK
II	16	71.01	701.7323 (+3)	701.7318	237-256	VNNSSLIGLGYTQTLKPGIK
III	16	65.17	1052.0958 (+2)	1052.0941	237-256	VNNSSLIGLGYTQTLKPGIK
I	17	66.00	947.5204 (+1)	947.5197	275-283	LGLGLEFQA
II	17	67.41	474.2634 (+2)	474.2635	275-283	LGLGLEFQA
III	17	65.52	947.5212 (+1)	947.5197	275-283	LGLGLEFQA

*A: N-terminal acetylated; ***C***: cysteine carboxyamidomethylated.

Table S6. Tryptic peptides found in the analysis of VDAC1 from NSC34-SOD1WT cell line after DTT reduction and carboxyamidomethylation. Retention time, experimentally measured and calculated monoisotopic m/z of the molecular ions, position in the sequence and peptide sequence of fragments present in the tryptic digest analyzed in triplicate of reduced and carboxyamidomethylated VDAC1 are reported. All sequences were confirmed by MS/MS. These sequences were used to build the sequence coverage reported in Figure 1.

Technical replicate	Frag. n.	Rt (min)	Monoisotopic m/z		Position in the sequence	Peptide sequence
			Measured	Calculated		
I	1	64.19	744.3967 (+2)	744.3968	2-15	*AVPPTYADLGKSAR
III	1	58.40	744.3966 (+2)	744.3968	2-15	*AVPPTYADLGKSAR
I	2	69.08	924.9798 (+2)	924.9782	2-18	*AVPPTYADLGKSARDVF
II	2	73.88	924.9789 (+2)	924.9782	2-18	*AVPPTYADLGKSARDVF
III	2	72.80	924.9794 (+2)	924.9782	2-18	*AVPPTYADLGKSARDVF
II	3	69.73	409.2302 (+2)	409.2305	8-15	ADLGKSAR
I	4	73.15	554.2939 (+2)	554.2938	9-18	DLGKSARDVF
II	4	74.64	554.2937 (+2)	554.2938	9-18	DLGKSARDVF
III	4	68.79	554.2928 (+2)	554.2938	9-18	DLGKSARDVF
III	5	27.07	552.7982 (+2)	552.7990	30-39	DLKTKSENGL
I	6	72.96	690.8546 (+2)	690.8545	30-41	DLKTKSENGLEF
II	6	74.32	690.8547 (+2)	690.8545	30-41	DLKTKSENGLEF
III	6	68.77	690.8539 (+2)	690.8545	30-41	DLKTKSENGLEF
I	7	69.40	725.6860 (+3)	725.6852	42-62	TSSGSANTETTKVNGSLETKY
II	7	70.13	725.6859 (+3)	725.6852	42-62	TSSGSANTETTKVNGSLETKY
III	7	64.78	725.6860 (+3)	725.6852	42-62	TSSGSANTETTKVNGSLETKY
III	8	26.66	569.8087 (+2)	569.8093	53-62	KVNGSLETKY
II	9	67.67	505.7617 (+2)	505.7618	54-62	VNGSLETKY
III	9	32.64	505.7614 (+2)	505.7618	54-62	VNGSLETKY
I	10	64.69	995.4905 (+2)	995.4904	76-93	NTDNTLGTEITVEDQLAR
II	10	67.94	995.4907 (+2)	995.4904	76-93	NTDNTLGTEITVEDQLAR
III	10	61.45	995.4908 (+2)	995.4904	76-93	NTDNTLGTEITVEDQLAR
I	11	72.68	1080.5433 (+2)	1080.5431	76-95	NTDNTLGTEITVEDQLARGL
II	11	73.75	1080.5432 (+2)	1080.5431	76-95	NTDNTLGTEITVEDQLARGL
III	11	69.15	1080.5435 (+2)	1080.5431	76-95	NTDNTLGTEITVEDQLARGL
I	12	63.82	666.3444 (+2)	666.3443	82-93	GTEITVEDQLAR
II	12	67.74	666.3445 (+2)	666.3443	82-93	GTEITVEDQLAR
III	12	57.19	666.3444 (+2)	666.3443	82-93	GTEITVEDQLAR
I	13	71.05	751.3967 (+2)	751.3970	82-95	GTEITVEDQLARGL
III	13	67.33	751.3962 (+2)	751.3970	82-95	GTEITVEDQLARGL
I	14	67.48	621.8030 (+2)	621.8042	96-106	KLTFDSSFSPN

I	15	74.44	536.3022 (+3)	536.3024	104-118	SPNTGKKNAKIKTGY
II	15	75.93	536.3016 (+3)	536.3024	104-118	SPNTGKKNAKIKTGY
I	16	74.75	436.9262 (+3)	436.9265	107-118	TGKKNAKIKTGY
II	16	76.10	436.9259 (+3)	436.9265	107-118	TGKKNAKIKTGY
III	16	20.28	436.9257 (+3)	436.9265	107-118	TGKKNAKIKTGY
I	17	36.14	414.7321 (+2)	414.7329	132-139	DIAGPSIR
II	17	67.74	414.7326 (+2)	414.7329	132-139	DIAGPSIR
III	17	36.44	414.7321 (+2)	414.7329	132-139	DIAGPSIR
I	18	65.31	535.3040 (+2)	535.3042	132-142	DIAGPSIRGAL
II	18	68.43	535.3028 (+2)	535.3042	132-142	DIAGPSIRGAL
III	18	62.33	535.3039 (+2)	535.3042	132-142	DIAGPSIRGAL
III	19	63.16	823.3996 (+1)	823.3990	143-149	VLGYEGW
I	20	71.91	648.8310 (+2)	648.8313	156-166	NFETSKSRVTQ
II	20	73.52	646.8308 (+2)	648.8313	156-166	NFETSKSRVTQ
I	21	72.98	822.9064 (+2)	822.9030	156-169	NFETSKSRVTQSNF
II	21	74.09	822.9046 (+2)	822.9030	156-169	NFETSKSRVTQSNF
III	21	68.04	822.9000 (+2)	822.9030	156-169	NFETSKSRVTQSNF
I	22	73.00	692.3475 (+2)	692.3473	158-169	ETSKSRVTQSNF
II	22	73.82	692.3474 (+2)	692.3473	158-169	ETSKSRVTQSNF
III	22	67.79	692.3484 (+2)	692.3473	158-169	ETSKSRVTQSNF
I	23	63.74	469.7383 (+2)	469.7387	162-169	SRVTQSNF
III	24	53.80	679.3043 (+2)	679.3051	179-190	**Q**LHTNVNDGTEF
I	25	64.12	687.8187 (+2)	687.8184	179-190	QLHTNVNDGTEF
II	25	67.91	687.8190 (+2)	687.8184	179-190	QLHTNVNDGTEF
III	25	57.72	687.8184 (+2)	687.8184	179-190	QLHTNVNDGTEF
II	26	61.52	917.9172 (+2)	917.9163	179-195	**Q**LHTNVNDGTEFGGSIY
III	26	59.82	917.9157 (+2)	917.9163	179-195	**Q**LHTNVNDGTEFGGSIY
I	27	32.37	567.2466 (+2)	567.2471	181-190	HTNVNDGTEF
III	27	32.70	567.2461 (+2)	567.2471	181-190	HTNVNDGTEF
II	28	42.18	498.7168 (+2)	498.7176	182-190	TNVNDGTEF
III	28	42.07	996.4284 (+1)	996.4274	182-190	TNVNDGTEF
I	29	73.07	734.4308 (+2)	734.4306	196-208	**Q**KVNKKLETAVNL
II	29	74.45	489.2330 (+3)	489.9564	196-208	**Q**KVNKKLETAVNL
III	29	68.81	489.9558 (+3)	489.9564	196-208	**Q**KVNKKLETAVNL

II	30	75.58	495.6315 (+3)	495.6319	196-208	QKVNKKLETAVNL
II	31	76.74	581.3372 (+3)	581.3374	196-210	QKVNKKLETAVNLAW
I	32	73.18	508.3111 (+2)	508.3115	200-208	KKLETAVNL
II	32	74.58	508.3115 (+2)	508.3115	200-208	KKLETAVNL
III	32	68.99	508.3107 (+2)	508.3115	200-208	KKLETAVNL
II	33	68.40	444.2637 (+2)	444.2640	201-208	KLETAVNL
I	34	64.63	612.7919 (+2)	612.7919	209-219	AWTAGNSNTRF
III	34	59.94	612.7917 (+2)	612.7919	209-219	AWTAGNSNTRF
II	35	67.60	484.2320 (+2)	484.2338	211-219	TAGNSNTRF
I	36	64.29	934.3621 (+1)	934.3616	226-233	**Q**VDPDA***C***F
II	36	64.19	934.3624 (+1)	934.3616	226-233	**Q**VDPDA***C***F
III	36	64.44	934.3624 (+1)	934.3616	226-233	**Q**VDPDA***C***F
I	37	48.78	476.1977 (+2)	476.1980	226-233	QVDPDA***C***F
II	37	48.24	476.1973 (+2)	476.1980	226-233	QVDPDA***C***F
III	37	48.61	951.3882 (+1)	952.3882	226-233	QVDPDA***C***F
I	38	70.25	601.8409 (+2)	601.8412	234-245	SAKVNNSSLIGL
II	38	71.69	601.8405 (+2)	601.8412	234-245	SAKVNNSSLIGL
III	38	66.57	601.8412 (+2)	601.8412	234-245	SAKVNNSSLIGL
I	39	70.16	711.8835 (+2)	711.8835	234-247	SAKVNNSSLIGLGY
II	39	71.23	711.8839 (+2)	711.8835	234-247	SAKVNNSSLIGLGY
III	39	66.78	711.8838 (+2)	711.8835	234-247	SAKVNNSSLIGLGY
I	40	73.26	549.8480 (+2)	549.8482	248-257	TQTLKPGIKL
II	40	74.66	549.8480 (+2)	549.8482	248-257	TQTLKPGIKL
III	40	69.19	549.8480 (+2)	549.8482	248-257	TQTLKPGIKL
III	41	69.32	435.2944 (+2)	435.2951	250-257	TLKPGIKL
I	42	73.51	435.2943 (+2)	435.2951	252-259	KPGIKLTL
II	42	74.89	435.2943 (+2)	435.2951	252-259	KPGIKLTL
I	43	74.04	531.9628 (+3)	531.9623	260-275	SALLDGKNVNAGGHKL
I	44	73.13	403.8825 (+3)	403.8832	264-275	DGKNVNAGGHKL
II	44	74.56	403.8827 (+3)	403.8832	264-275	DGKNVNAGGHKL

*A: N-terminal acetylated; ***C***: cysteine carboxyamidomethylated; **Q**: pyroglutamic acid form.

Table S7. Chymotryptic peptides found in the analysis of VDAC1 from NSC34-SOD1WT cell line after DTT reduction and carboxyamidomethylation. Retention time, experimentally measured and calculated monoisotopic m/z of the molecular ions, position in the sequence and peptide sequence of fragments present in the chymotryptic digest analyzed in triplicate of reduced and carboxyamidomethylated VDAC1 are reported. All sequences were confirmed by MS/MS. These sequences were used to build the sequence coverage reported in Figure 1.

Frag. n.	Technical replicate	Rt (min)	Monoisotopic m/z		Position in the sequence	Peptide sequence
			Measured	Calculated		
1	I	68.03	1059.9933 (+2)	1059.9919	121-139	EHINLG**C**DVDFDIAGPSIR
	II	69.12	1059.9924 (+2)			
	III	64.33	1059.9928 (+2)			
2	I	71.27	1225.5894 (+2)	1225.5888	140-161	GALVLGYEGWLAGYQ**M**NFETSK
	II	72.76	1225.5894 (+3)			
	III	69.23	817.3956 (+3)	817.3951		
3	I	69.70	1233.5853 (+2)	1233.5862	140-161	GALVLGYEGWLAGYQ**M**NFETSK
	II	71.59	1233.5875 (+2)			
	III	66.90	1233.5859 (+2)			

C: cysteine oxidized to sulfonic acid; **M**: methionine sulfoxide; **M**: methionine sulfone.

Table S8. Retention time, experimentally measured and calculated monoisotopic m/z of the molecular ions, position in the sequence and peptide sequence of sulfur containing tryptic fragments found in the analysis of VDAC1 from NSC34-SOD1WT cell line digest reduced with DTT, carboxyamidomethylated and digested in-solution. Fragment 1 was used to build the sequence coverage reported in Figure 1.

Frag. n.	Technical replicate	Rt (min)	Monoisotopic m/z		Position in the sequence	Peptide sequence	MS/MS
			Measured	Calculated			
1	I	73.53	531.9159 (+3)	531.9161	119-131	KREHINLG**C**DVDF	Yes
	II	74.94	531.9160 (+3)	531.9161	119-131	KREHINLG**C**DVDF	Yes
	III	79.52	531.9161 (+3)	531.9161	119-131	KREHINLG**C**DVDF	No

C: cysteine oxidized to sulfonic acid.

Table S9. Retention time, experimentally measured and calculated monoisotopic m/z of the molecular ion of the chymotryptic peptide K^{119}REHINLG**C**DVDF^{131}found in the analysis of VDAC1 from NSC34-SOD1WT cell line digest reduced with DTT, carboxyamidomethylated and digested in-solution. This fragment was used to build the sequence coverage reported in Figure 1.

Technical replicate	Peptide	Measured monoisotopic *m*/*z*	Absolute intensity	Ratio Ox/Red
I	GALVLGYEGWLAGYQ**M**NFETSK	1225.5894 (+2)	$2.4 \cdot 10^{6}$	2.6
I	GALVLGYEGWLAGYQMNFETSK	1217.5923 (+2)	$9.3 \cdot 10^{5}$	
II	GALVLGYEGWLAGYQ**M**NFETSK	1225.5894 (+3)	$3.6 \cdot 10^{6}$	2.8
II	GALVLGYEGWLAGYQMNFETSK	1217.5917 (+2)	$1.3 \cdot 10^{6}$	
III	GALVLGYEGWLAGYQ**M**NFETSK	817.3956 (+3)	$1.7 \cdot 10^{6}$	3.7
III	GALVLGYEGWLAGYQMNFETSK	812.0640 (+3)	$4.6 \cdot 10^{5}$	
I	GALVLGYEGWLAGYQ**M**NFETSK	1233.5853 (+2)	$6.4 \cdot 10^{4}$	0.07
I	GALVLGYEGWLAGYQMNFETSK	1217.5923 (+2)	$9.3 \cdot 10^{5}$	
II	GALVLGYEGWLAGYQ**M**NFETSK	1233.5875 (+2)	$5.1 \cdot 10^{4}$	0.04
II	GALVLGYEGWLAGYQMNFETSK	1217.5917 (+2)	$1.3 \cdot 10^{6}$	
III	GALVLGYEGWLAGYQ**M**NFETSK	1233.5859 (+2)	$3.9 \cdot 10^{4}$	0.09
III	GALVLGYEGWLAGYQMNFETSK	812.0640 (+3)	$4.6 \cdot 10^{5}$	

M: methionine sulfoxide; **M**: methionine sulfone.

Table S10. Ratio of the absolute intensities of the molecular ions of the sulfur and not sulfur containing tryptic peptide G^{140}ALVLGYEGWLAGYQMNFETSK^{161} found in the analysis of VDAC1 from NSC34-SOD1WT cell line digest reduced with DTT, carboxyamidomethylated and digested in-solution.

Technical replicate	Frag. n.	Rt (min)	Monoisotopic m/z		Position in the sequence	Peptide sequence
			Measured	Calculated		
I	1	55.09	587.3113 (+2)	587.3111	2-12	*AVPPTYADLGK
II	1	55.51	587.3112 (+2)	587.3111	2-12	*AVPPTYADLGK
III	1	54.84	587.3118 (+2)	587.3111	2-12	*AVPPTYADLGK
I	2	70.05	854.4777 (+1)	854.4771	21-28	GYGFGLIK
II	2	69.44	854.4772 (+1)	854.4771	21-28	GYGFGLIK
III	2	52.56	854.4739 (+1)	854.4771	21-28	GYGFGLIK
I	3	66.43	730.3458 (+3)	730.3450	33-53	TKSENGLEFTSSGSANTETTK
II	3	66.64	730.3455 (+3)	730.3450	33-53	TKSENGLEFTSSGSANTETTK
III	3	32.44	730.3464 (+3)	730.3450	33-53	TKSENGLEFTSSGSANTETTK
I	4	57.97	980.4425 (+2)	980.4425	35-53	SENGLEFTSSGSANTETTK
II	4	54.21	980.4446 (+2)	980.4425	35-53	SENGLEFTSSGSANTETTK
III	4	39.68	980.4435 (+2)	980.4425	35-53	SENGLEFTSSGSANTETTK
I	5	69.33	687.8326 (+2)	687.8324	64-74	WTEYGLTFTEK
II	5	68.92	687.8330 (+2)	687.8324	64-74	WTEYGLTFTEK
III	5	54.62	687.8333 (+2)	687.8324	64-74	WTEYGLTFTEK
I	6	68.40	1088.5283 (+2)	1088.5295	75-93	WNTDNTLGTEITVEDQLAR
II	6	67.72	1088.5300 (+2)	1088.5295	75-93	WNTDNTLGTEITVEDQLAR
III	6	62.37	1088.5308 (+2)	1088.5295	75-93	WNTDNTLGTEITVEDQLAR
I	7	61.95	700.8390 (+2)	700.8383	97-109	LTFDSSFSPNTGK
II	7	61.64	700.8387 (+2)	700.8383	97-109	LTFDSSFSPNTGK
III	7	48.36	700.8392 (+2)	700.8383	97-109	LTFDSSFSPNTGK
I	8	66.91	764.8867 (+2)	764.8857	97-110	LTFDSSFSPNTGKK
II	8	69.31	764.8865 (+2)	764.8857	97-110	LTFDSSFSPNTGKK
III	8	41.88	510.2604 (+3)	510.2596	97-110	LTFDSSFSPNTGKK
I	9	69.01	710.0155 (+3)	710.0091	121-139	EHINLG*C*DVDFDIAGPSIR (no MS/MS)
II	9	68.70	710.0140 (+3)	710.0091	121-139	EHINLG*C*DVDFDIAGPSIR
III	9	67.38	710.0140 (+3)	710.0091	121-139	EHINLG*C*DVDFDIAGPSIR
I	10	73.18	1217.5931 (+2)	1217.5910	140-161	GALVLGYEGWLAGYQMNFETSK (no MS/MS)
II	10	72.95	1217.5940 (+2)	1217.5910	140-161	GALVLGYEGWLAGYQMNFETSK

III	10	70.09	812.0656 (+3)	812.0631	140-161	GALVLGYEGWLAGYQMNFETSK
I	11	59.56	607.3142 (+2)	607.3142	164-174	VTQSNFAVGYK
II	11	58.93	607.3145 (+2)	607.3142	164-174	VTQSNFAVGYK
III	11	39.20	607.3148 (+2)	607.3142	164-174	VTQSNFAVGYK
I	12	69.75	867.4030 (+3)	867.4015	175-197	TDEFQLHTNVNDGTEFGGSIYQK
II	12	69.24	867.4023 (+3)	867.4015	175-197	TDEFQLHTNVNDGTEFGGSIYQK
III	12	62.81	867.4026 (+3)	867.4015	175-197	TDEFQLHTNVNDGTEFGGSIYQK
I	13	70.28	649.3417 (+3)	649.3412	201-218	KLETAVNLAWTAGNSNTR
II	13	69.85	973.5087 (+2)	973.5081	201-218	KLETAVNLAWTAGNSNTR
III	13	63.75	649.3419 (+3)	649.3412	201-218	KLETAVNLAWTAGNSNTR
I	14	64.81	909.4618 (+2)	909.4607	202-218	LETAVNLAWTAGNSNTR
II	14	64.37	909.4613 (+2)	909.4607	202-218	LETAVNLAWTAGNSNTR
III	14	53.21	909.4614 (+2)	909.4607	202-218	LETAVNLAWTAGNSNTR
I	15	59.52	700.8118 (+2)	700.8112	225-236	YQVDPDA***C***FSAK
II	15	58.69	700.8120 (+2)	700.8112	225-236	YQVDPDA***C***FSAK
III	15	42.95	700.8119 (+2)	700.8112	225-236	YQVDPDA***C***FSAK
III	16	54.09	854.4678 (+2)	854.4680	237-252	VNNSSLIGLGYTQTLK
I	17	70.56	701.7321 (+3)	701.7318	237-256	VNNSSLIGLGYTQTLKPGIK
II	17	70.19	1052.0951 (+2)	1052.0941	237-256	VNNSSLIGLGYTQTLKPGIK
III	17	64.62	1052.0957 (+2)	1052.0941	237-256	VNNSSLIGLGYTQTLKPGIK
III	18	47.48	603.3399 (+3)	603.3390	257-274	LTLSALLDGKNVNAGGHK
I	19	66.80	947.5209 (+1)	947.5197	275-283	LGLGLEFQA
II	19	66.75	474.2632 (+2)	474.2635	275-283	LGLGLEFQA
III	19	65.30	947.5202 (+1)	947.5197	275-283	LGLGLEFQA

*A: N-terminal acetylated; ***C***: cysteine carboxyamidomethylated.

Table S11. Tryptic peptides found in the analysis ofVDAC1 from NSC34-SOD1G93A cell line after DTT reduction and carboxyamidomethylation. Retention time, experimentally measured and calculated monoisotopic m/z of the molecular ions, position in the sequence and peptide sequence of fragments present in the tryptic digest analyzed in triplicate of reduced and carboxyamidomethylated VDAC1 are reported. All sequences were confirmed by MS/MS. These sequences were used to build the sequence coverage reported in Figure 1.

Technical replicate	Frag. n.	Rt (min)	Monoisotopic m/z		Position in the sequence	Peptide sequence
			Measured	Calculated		
I	1	88.07	924.9806 (+2)	924.9782	2-18	*AVPPTYADLGKSARDVF
II	1	83.21	924.9793 (+2)	924.9782	2-18	*AVPPTYADLGKSARDVF
III	1	82.81	924.9792 (+2)	924.9782	2-18	*AVPPTYADLGKSARDVF
I	2	88.50	690.8545 (+2)	690.8545	30-41	DLKTKSENGLEF
II	2	83.94	690.8542 (+2)	690.8545	30-41	DLKTKSENGLEF
III	2	83.82	690.8547 (+2)	690.8545	30-41	DLKTKSENGLEF
I	3	87.95	725.6855 (+3)	725.6852	42-62	TSSGSANTETTKVNGSLETKY
II	3	83.11	1088.0251 (+2)	1088.0248	42-62	TSSGSANTETTKVNGSLETKY
III	3	82.68	725.6858 (+3)	725.6852	42-62	TSSGSANTETTKVNGSLETKY
I	4	79.99	995.4911 (+2)	995.4904	76-93	NTDNTLGTEITVEDQLAR
II	4	80.47	995.4907 (+2)	995.4904	76-93	NTDNTLGTEITVEDQLAR
III	4	79.96	995.4906 (+2)	995.4904	76-93	NTDNTLGTEITVEDQLAR
I	5	87.73	1080.5437 (+2)	1080.5431	76-95	NTDNTLGTEITVEDQLARGL
II	5	83.16	1080.5441 (+2)	1080.5431	76-95	NTDNTLGTEITVEDQLARGL
III	5	82.76	1080.5439 (+2)	1080.5431	76-95	NTDNTLGTEITVEDQLARGL
I	6	52.73	522.7746 (+2)	522.7751	82-91	GTEITVEDQL
II	7	83.41	472.7396 (+2)	472.7404	96-103	KLTFDSSF
III	8	84.48	536.3029 (+3)	536.3024	104-118	SPNTGKKNAKIKTGY
II	9	84.51	436.9258 (+3)	436.9265	107-118	TGKKNAKIKTGY
III	9	84.54	436.9255 (+3)	436.9265	107-118	TGKKNAKIKTGY
I	10	80.22	414.7322 (+2)	414.7329	132-139	DIAGPSIR
III	10	80.44	414.7325 (+2)	414.7329	132-139	DIAGPSIR
I	11	88.12	535.3046 (+2)	535.3042	132-142	DIAGPSIRGAL
II	11	83.16	535.3044 (+2)	535.3042	132-142	DIAGPSIRGAL
III	11	82.77	535.3043 (+2)	535.3042	132-142	DIAGPSIRGAL
I	12	70.37	823.3982 (+1)	823.3990	143-149	VLGYEGW
III	12	70.69	823.3976 (+1)	823.3990	143-149	VLGYEGW
I	13	88.44	648.8308 (+2)	648.8313	156-166	NFETSKSRVTQ
II	13	83.90	648.8313 (+2)	648.8313	156-166	NFETSKSRVTQ
I	14	88.50	548.9374 (+3)	548.9380	156-169	NFETSKSRVTQSNF

II	14	84.12	548.9379 (+3)	548.9380	156-169	NFETSKSRVTQSNF
III	14	84.02	548.9378 (+3)	548.9380	156-169	NFETSKSRVTQSNF
II	15	83.95	692.3478 (+2)	692.3473	158-169	ETSKSRVTQSNF
III	15	83.77	692.3476 (+2)	692.3473	158-169	ETSKSRVTQSNF
II	16	84.12	665.6419 (+3)	665.6418	174-190	KTDEFQLHTNVNDGTEF
III	16	84.02	665.6417 (+3)	665.6418	174-190	KTDEFQLHTNVNDGTEF
I	17	88.50	824.7165 (+3)	824.7158	174-195	KTDEFQLHTNVNDGTEFGGSIY
II	17	84.16	824.7150 (+3)	824.7158	174-195	KTDEFQLHTNVNDGTEFGGSIY
III	17	84.05	824.7164 (+3)	824.7158	174-195	KTDEFQLHTNVNDGTEFGGSIY
I	18	86.26	687.8181 (+2)	687.8184	179-190	QLHTNVNDGTEF
II	18	83.05	687.8186 (+2)	687.8184	179-190	QLHTNVNDGTEF
III	18	82.63	687.8184 (+2)	687.8184	179-190	QLHTNVNDGTEF
I	19	86.87	926.4302 (+2)	926.4296	179-195	QLHTNVNDGTEFGGSIY
II	19	83.11	926.4309 (+2)	926.4296	179-195	QLHTNVNDGTEFGGSIY
III	19	82.67	926.4287 (+2)	926.4296	179-195	QLHTNVNDGTEFGGSIY
I	20	41.55	996.4279 (+1)	996.4274	182-190	TNVNDGTEF
I	21	88.66	489.9559 (+3)	489.9564	196-208	**Q**KVNKKLETAVNL
II	21	84.16	489.9561 (+3)	489.9564	196-208	**Q**KVNKKLETAVNL
III	21	84.08	489.9560 (+3)	489.9564	196-208	**Q**KVNKKLETAVNL
III	22	84.39	495.6313 (+3)	495.6319	196-208	QKVNKKLETAVNL
I	23	87.95	612.7919 (+2)	612.7919	209-219	AWTAGNSNTRF
III	23	82.67	612.7914 (+2)	612.7919	209-219	AWTAGNSNTRF
II	24	81.53	484.2330 (+2)	484.2338	211-219	TAGNSNTRF
III	24	80.99	484.2330 (+2)	484.2338	211-219	TAGNSNTRF
I	25	47.41	476.1971 (+2)	476.1980	226-233	QVDPDA**C**F
II	25	47.88	476.1969 (+2)	476.1980	226-233	QVDPDA**C**F
III	25	48.78	476.1981 (+2)	476.1980	226-233	QVDPDA**C**F
I	26	88.02	601.8414 (+2)	601.8412	234-245	SAKVNNSSLIGL
II	26	83.16	601.8420 (+2)	601.8412	234-245	SAKVNNSSLIGL
III	26	82.77	601.8409 (+2)	601.8412	234-245	SAKVNNSSLIGL
II	27	83.19	711.8846 (+2)	711.8835	234-247	SAKVNNSSLIGLGY
III	27	82.77	711.8849 (+2)	711.8835	234-247	SAKVNNSSLIGLGY
I	28	88.75	549.8484 (+2)	549.8482	248-257	TQTLKPGIKL
II	28	84.25	549.8483 (+2)	549.8482	248-257	TQTLKPGIKL

III	28	84.17	549.8484 (+2)	549.8482	248-257	TQTLKPGIKL
I	29	88.80	435.2948 (+2)	435.2951	250-257	TLKPGIKL
II	29	84.29	435.2946 (+2)	435.2951	250-257	TLKPGIKL
I	30	91.98	627.4189 (+2)	627.4138	252-263	KPGIKLTLSALL

*A: N-terminal acetylated; ***C***: cysteine carboxyamidomethylated; **Q**: pyroglutamic acid form.

Table S12. Chymotryptic peptides found in the analysis of VDAC1 from NSC34-SOD1G93A cell line after DTT reduction and carboxyamidomethylation. Retention time, experimentally measured and calculated monoisotopic m/z of the molecular ions, position in the sequence and peptide sequence of fragments present in the chymotryptic digest analyzed in triplicate of reduced and carboxyamidomethylated VDAC1 are reported. All sequences were confirmed by MS/MS. These sequences were used to build the sequence coverage reported in Figure 1.

Frag. n.	Technical replicate	Rt (min)	Monoisotopic m/z		Position in the sequence	Peptide sequence
			Measured	Calculated		
1	I	69.21	1059.9921 (+2)	1059.9919	121-139	EHINLG**C**DVDFDIAGPSIR
	II	68.72	1059.9915 (+2)			
	III	63.35	1059.9928 (+2)			
2	I	72.24	1225.5892 (+2)	1225.5888	140-161	GALVLGYEGWLAGYQ**M**NFETSK
	II	71.95	1225.5897 (+2)			
	III	68.76	817.3946 (+3)	817.3951		
3	I	71.06	822.7261 (+3)	822.7268	140-161	GALVLGYEGWLAGYQ**M**NFETSK
	II	70.64	822.7269 (+3)			
	III	66.11	822.7277 (+3)			

C: cysteine oxidized to sulfonic acid; **M**: methionine sulfoxide; **M**: methionine sulfone.

Table S13. Retention time, experimentally measured and calculated monoisotopic m/z of the molecular ions, position in the sequence and peptide sequence of sulfur containing tryptic fragments found in VDAC1 from NSC34-SOD1 G93A cell line digest reduced with DTT, carboxyamidomethylated and digested in-solution. Fragment 1 was used to build the sequence coverage reported in Figure 1.

Technical triplicate	Rt (min)	Monoisotopic m/z	
		Measured	Calculated
I	66.49	804.8686 (+2)	804.8692
II	68.19	804.8689 (+2)	
III	60.00	804.8690 (+2)	

Table S14. Retention time, experimentally measured and calculated monoisotopic m/z of the molecular ion of the phosphorylated tryptic peptide L^{97}TFDSSF**S**PNTGKK^{110} containing the Ser^{104} phosphorylated found in VDAC1 from NSC34 cell line digest reduced with DTT, carboxyamidomethylated and digested in-solution. The sequence was confirmed by MS/MS.

Technical replicate	Rt (min)	Monoisotopic m/z	
		Measured	Calculated
I	61.08	804.8690 (+2)	804.8692
II	61.62	804.8699 (+2)	
III	57.05	804.8701 (+2)	

Table S15. Retention time, experimentally measured and calculated monoisotopic m/z of the molecular ion of the phosphorylated tryptic peptide L^{97}TFDSSF**S**PNTGKK^{110} containing the Ser^{104} phosphorylated found in VDAC1 from NSC34-SOD1 cell line digest reduced with DTT, carboxyamidomethylated and digested in-solution. The sequence was confirmed by MS/MS.

Technical replicate	Rt (min)	Monoisotopic m/z	
		Measured	Calculated
I	61.78	804.8701 (+2)	804.8692
II	61.06	804.8702 (+2)	
III	45.63	536.9153 (+3)	536.9154

Table S16. Retention time, experimentally measured and calculated monoisotopic m/z of the molecular ion of the phosphorylated tryptic peptide L^{97}TFDSSF**S**PNTGKK^{110} containing the Ser^{104} phosphorylated found in VDAC1 from NSC34-SOD1G93A cell line digest reduced with DTT, carboxyamidomethylated and digested in-solution. The sequence was confirmed by MS/MS.

Technical replicate	Peptide	Measured monoisotopic *m/z*	Absolute intensity	Ratio Phospho/Norm
I	LTFDSSF**S**PNTGKK	804.8686 (+2)	$1.2 \cdot 10^{5}$	0.08
I	LTFDSSFSPNTGKK	764.8865 (+2)	$1.5 \cdot 10^{6}$	
II	LTFDSSF**S**PNTGKK	804.8689 (+2)	$2.2 \cdot 10^{5}$	0.01
II	LTFDSSFSPNTGKK	510.2602 (+3)	$2.0 \cdot 10^{7}$	
III	LTFDSSF**S**PNTGKK	804.8690 (+2)	$2.8 \cdot 10^{4}$	0.03
III	LTFDSSFSPNTGKK	764.8865 (+2)	$1.0 \cdot 10^{6}$	

S: serine phosphorylated.

Table S17. Ratio of the absolute intensities of the molecular ions of the phosphorylated and not phosphorylated tryptic peptide L^{97}TFDSSF**S**PNTGKK^{110} containing the Ser^{104} phosphorylated found in the analysis of VDAC1 from Mouse Motor Neuron-like NSC34 cell line digest reduced with DTT, carboxyamidomethylated and digested in-solution.

Technical replicate	Peptide	Measured monoisotopic *m/z*	Absolute intensity	Ratio Phospho/Norm
I	LTFDSSF**S**PNTGKK	804.8690 (+2)	$4.4 \cdot 10^{4}$	0.03
I	LTFDSSFSPNTGKK	764.8859 (+2)	$1.7 \cdot 10^{6}$	
II	LTFDSSF**S**PNTGKK	804.8699 (+2)	$9.0 \cdot 10^{4}$	0.05
II	LTFDSSFSPNTGKK	764.8865 (+2)	$1.9 \cdot 10^{6}$	
III	LTFDSSF**S**PNTGKK	804.8701 (+2)	$2.8 \cdot 10^{4}$	0.03
III	LTFDSSFSPNTGKK	764.8866 (+2)	$1.1 \cdot 10^{6}$	

S: serine phosphorylated.

Table S18. Ratio of the absolute intensities of the molecular ions of the phosphorylated and not phosphorylated tryptic peptide L^{97}TFDSSF**S**PNTGKK^{110}containing the Ser^{104} phosphorylated found in the analysis of VDAC1 from Mouse Motor Neuron-like NSC34-SOD1WT cell line digest reduced with DTT, carboxyamidomethylated and digested in-solution.

Technical replicate	Peptide	Measured monoisotopic *m*/*z*	Absolute intensity	Ratio Phospho/Norm
I	LTFDSSF**S**PNTGKK	804.8701 (+2)	$9.6 \cdot 10^4$	0.06
I	LTFDSSFSPNTGKK	764.8866 (+2)	$1.6 \cdot 10^6$	
II	LTFDSSF**S**PNTGKK	804.8702 (+2)	$7.3 \cdot 10^4$	0.05
II	LTFDSSFSPNTGKK	764.8866 (+2)	$1.4 \cdot 10^6$	
III	LTFDSSF**S**PNTGKK	536.9153 (+3)	$5.4 \cdot 10^5$	0.02
III	LTFDSSFSPNTGKK	510.2602 (+3)	$3.3 \cdot 10^7$	

S: serine phosphorylated.

Table S19. Ratio of the absolute intensities of the molecular ions of the phosphorylated and not phosphorylated tryptic peptide L^{97}TFDSSF**S**PNTGKK^{110} containing the Ser^{104} phosphorylated found in the analysis of VDAC1 from Mouse Motor Neuron-like NSC34-SOD1G93A cell line digest reduced with DTT, carboxyamidomethylated and digested in-solution.

Frag. n.	Rt (min)	Monoisotopic m/z		Position in the sequence	Peptide sequence
		Measured	Calculated		
1	34.22	730.6730 (+3)	730.6734	33-53	TKSE**N**GLEFTSSGSANTETTK
2	40.30	654.2921 (+3)	654.2925	35-53	SE**N**GLEFTSSGSANTETTK
3	44.12	510.5876 (+3)	510.5880	97-110	LTFDSSFSP**N**TGKK
4	40.58	607.8062 (+2)	607.8065	164-174	VT**Q**SNFAVGYK
5	48.84	649.6692 (+3)	649.6696	201-218	KLETAV**N**LAWTAGNSNTR
6	54.76	909.9527(+2)	909.9530	202-218	LETAVNLAWTAG**N**SNTR
7	44.14	701.3032 (+2)	701.3035	225-236	Y**Q**VDPDA***C***FSAK
8	53.78	702.0598 (+3)	702.0602	237-256	VN**N**SSLIGLGYTQTLKPGIK

C: cysteine carboxyamidomethylated; **N**: asparagine deamidated; **Q**: glutamine deamidated.

Table S20. Retention time, experimentally measured and calculated monoisotopic m/z of the molecular ions, position in the sequence and peptide sequence of tryptic fragments containing asparagine and glutamine residues in deamidated form found in VDAC1 from NSC34-SOD1G93A cell line digest reduced with DTT, carboxyamidomethylated and digested in-solution. All sequences were confirmed by MS/MS.
